# Enrichment of Cysteine S-palmitoylated Peptides Using Sodium Deoxycholate Acid Precipitation

**DOI:** 10.1016/j.mcpro.2025.101218

**Published:** 2025-10-16

**Authors:** Peter T. Jensen, Giuseppe Palmisano, Christopher J. Rhodes, Martin R. Larsen

**Affiliations:** 1 Protein Research Group, Department of Biochemistry and Molecular Biology, University of Southern Denmark, Odense M, Denmark; 2 Glycoproteomics Laboratory, Department of Parasitology, ICB, University of São Paulo, São Paulo, São Paulo, Brazil; 3 Kovler Diabetes Center, Section of Endocrinology, Department of Medicine, University of Chicago, Chicago, Illinois, USA; 4 Early Cardiovascular, Renal and Metabolism, Biopharmaceuticals R&D, AstraZeneca, Gaithersburg, Maryland, USA

**Keywords:** S-palmitoylation, post-translational modification, mass spectrometry, sodium deoxycholate, mouse brain membrane proteins, diabetes, zDHHC proteins

## Abstract

S-palmitoylation is a poorly understood post-translational modification that is gaining more attention as an essential regulator of cellular processes. The reversible nature of S-palmitoylation may allow for fine-tuned control of cellular events and adaptation to stimuli. The detection of S-palmitoylated proteins and peptides includes the Acyl-Biotin Exchange (ABE) method, Acyl resin-assisted Capture (Acyl-RAC), metabolic labelling, and derivatives thereof. We present a novel method of enrichment of S-palmitoylated peptides termed *SDC Acid Precipitation Enrichment* (SDC-ACE). Here, S-palmitoylated peptides are enriched by taking advantage of their co-precipitation with Sodium deoxycholate (SDC) under acidic conditions, allowing easy and fast separation of lipidated peptides from the sample suspension. We initially applied our novel method for the characterization of the mouse brain, providing an in-depth analysis of S-palmitoylation events within the brain and comprehensive profile of the mouse brain S-palmitoylome. Further, we applied our method for mapping mouse tissue-specific S-palmitoylation, highlighting the extensive role of S-palmitoylation throughout various organs in the body. Finally, we applied our methods for studying the brain palmitoylome of diabetic *db/db* mouse, uncovering alterations in the palmitoylation of proteins associated with obesity and type 2 diabetes. The SDC-ACE method allows fast and easy enrichment of S-palmitoylated peptides, providing a valuable tool for exploring the dynamics and function of S-palmitoylation in diverse biological systems.

## Highlights

• Sodium Deoxycholate Acid Precipitation efficiently identify S-palmitoylation sites.
• SDC-ACE surpasses current methodologies for identifying S-palmitoylated sites.
• More than 33,000 formerly S-palmitoylated peptides were identified from mouse brain.
• SDC-ACE identify tissue-specific S-palmitoylation events in mice organs.
• S-palmitoylation is observed on enzymes involved in regulation of PTMs.
• SDC-ACE reveal regulated S-palmitoylated sites linked to obesity linked diabetes.

## Graphical Abstract

**Figure:**
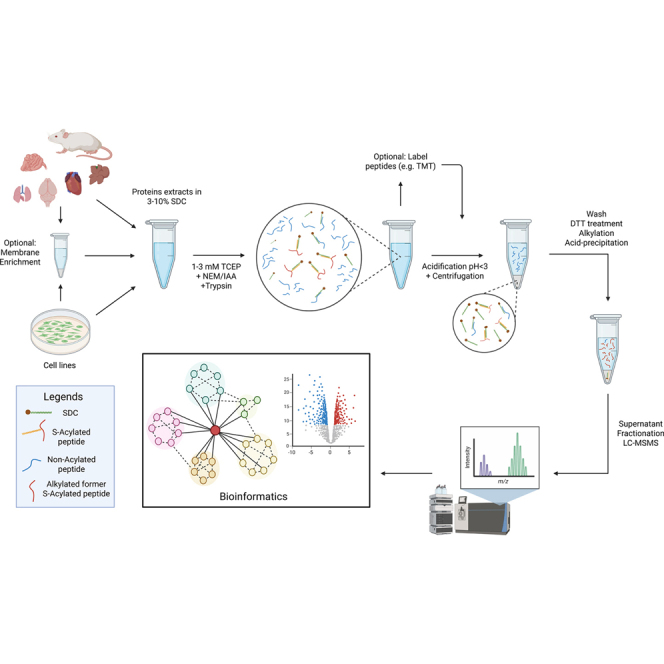

## In Brief

In this article, we present a novel simple and fast method for enriching and characterizing formerly S-palmitoylated peptides using sodium deoxycholate liquid–solid separation combined with acid precipitation and dithiothreitol reduction (termed the SDC-ACE method). The method surpasses current methodologies for assessing formerly S-palmitoylated peptides with respect to numbers, sensitivity and simplicity. It resulted in the identification of more than 33.000 formerly S-palmitoylated peptides from mouse brain membrane preparations and identification of tissue specific palmitoylation patterns.

Protein lipidation is an underappreciated post-translational modification (PTM) that plays critical roles in regulating protein location, function, and activity within cells. This process involves the covalent attachment of lipid molecules, such as fatty acids, to specific amino acids in proteins. The significance of protein lipidation lies in an ability to dynamically modulate various aspects of protein behavior, such as binding affinities, stability, folding patterns, cellular localization, and interaction with other macromolecules (1). Given its central role in numerous cellular functions—including trafficking, migration, and signal transduction—protein lipidation is a key consideration in biomedical research. Importantly, abnormalities in protein lipidation are linked to various diseases such as cancer (reviewed in (2)), neurodegenerative conditions (reviewed in (3)), and metabolic disorders (4, 5, 6), making protein lipidation an area of keen interest and highlighting a potential as a target for therapeutic intervention.

Among the different types of protein lipidation, cysteine S-acylation is the most prevalent. This modification involves a two-step enzymatic addition of a fatty acid moiety to the sulfur atom of cysteine residues in a protein via a thioester linkage (7, 8, 9). In contrast to most protein lipid modifications, S-acylation is reversible, allowing for the dynamic regulation of protein function, activity, and cellular localization, thereby making it a critical player in cellular signaling pathways and other physiological processes (10, 11). Although different long-chain acyl moieties can be conjugated to proteins’ appropriately exposed cysteine, S-acylation is more commonly referred to as S-palmitoylation (or palmitoylation) since most S-acylation modifications commonly utilize the 16-carbon saturated fatty acid, palmitate. The enzymes responsible for S-palmitoylation are protein acyltransferases (also referred to as palmitoyltransferases (PATs)), a family of 23 genes in humans (12) and 24 in mouse (13), also termed the S-palmitoylation “writers”. These enzymes are integral membrane proteins and feature a highly conserved aspartate-histidine-histidine-cysteine (DHHC) motif housed within a zinc-binding cysteine-rich domain (zDHHC), which is vital for their catalytic activity (7, 8, 9, 14, 15). Several zDHHC PATs are themselves S-palmitoylated, regulating their membrane association and activity (16, 17). One example is the regulatory interplay between zDHHC6 and zDHHC16, where the protein stability and catalytic activity of zDHHC6 is regulated through zDHHC16-catalyzed palmitoylation of three cysteines on the cytosolic C-terminal tail of zDHHC6 (16). In contrast to the relatively high number of S-palmitoylation “writers”, only a few proteins have been identified as S-palmitoylation “erasers”, including the Acyl-protein thioesterase 1 and 2 (APT1/2), protein palmitoyl thioesterases (PPTs) and members of the alpha/beta hydrolase domain-containing (ABHD) protein family (3). A dynamic interplay between S-palmitoylation “writers” and “erasers” accounts for the reversibility of this PTM, although the regulation of the process is not well-defined and likely to be particular to a given protein S-palmitoylation and related cellular function(s).

Studying S-palmitoylation is a complex analytical task due to the attachment of the fatty acid-modifying group, which binds to surfaces and chromatographic resin, complicating the isolation and identification of intact S-palmitoylated peptides. Therefore, most developed methods for studying S-palmitoylation sites in proteins rely on sequential reduction of the S-palmitoyl group combined with specialized affinity-based techniques, for the identification of formerly S-palmitoylated peptides using liquid chromatography coupled with tandem mass spectrometry (LC-MS/MS). These methods mostly include variations of Acyl-Biotin Exchange (ABE) (18, 19). ABE involves the complete alkylation of free cysteines on the target proteins, to avoid false positive identifications, followed by the removal of the S-palmitoyl group on cysteines using high molar concentrations of hydroxylamine (HA) (20). Then, the newly freed cysteine thiol groups are reacted with a thiol-reactive biotin molecule and subsequently, these biotinylated S-palmitoylated proteins are enriched by capture on streptavidin/avidin beads. This approach was first adapted by Roth *et al*. (21), for global analysis of protein S-palmitoylation. Releasing proteins from the biotin moiety, followed by cysteine alkylation, tryptic peptide digestion then LC-MS/MS can identify and quantify S-palmitoylated proteins in the sample. Yang *et al*., reported a different adaptation, named palmitoyl protein identification and site characterization (PalmPISC) (22). Here, using the ABE method with affinity enrichment of biotinylated S-palmitoylated proteins at the peptide-level, allowed for identification of 398 S-palmitoylated proteins and 168 S-palmitoylation sites, making the approach more suitable for larger-scale characterization of S-palmitoylated sites (22). Acyl resin-assisted capture (Acyl-RAC), developed by Forrester *et al*., is another affinity-based approach utilizing methanethiosulfonate (MMTS) to alkylate free cysteines followed by hydroxylamine treatment and capture of formerly S-palmitoylated cysteines with thiol-reactive thiopropyl Sepharose resin (23). Collins *et al*., further developed on the ABE approach, named site-specific ABE (ssABE), utilizing molecular weight-based cut-off concentration to reduce protein loss and eliminate labour-intensive protein precipitation (24). This ensured removal of chemical reagents, which can interfere with subsequent protein analysis (24). Using this approach, they identified 906 putative S-palmitoylation sites in 641 proteins from mouse forebrain.

For ABE, Acyl-RAC and other related methods that have been developed over the years, researchers have used either 100 mM HA or Tris buffer as a control, which theoretically does not release all the S-palmitoyl group from cysteines. As an alternative method to affinity-based techniques for characterization of S-palmitoylation, metabolic labelling approaches employing bio-orthogonal probes (25), such as 17-octadecyonic acid (17-ODYA) (26), has been used. The 17-ODYA-labeled sites can be subsequently identified using click chemistry with biotin-probes combined with LC-MS/MS analysis, immunoblotting, and/or imaging (e.g., (26)).

Here we present a simple and fast method for enriching and characterizing formerly S-palmitoylated peptides using sodium deoxycholate (SDC) liquid-solid separation combined with acid precipitation and dithiothreitol (DTT) reduction. We have termed this more convenient approach SDC Acid Precipitation Enrichment (SDC-ACE). We optimized the method on synthetic S-palmitoylated peptides and applied it to different biological settings, including mouse organs and obesity-linked diabetic mouse brains. In total, we identified more than 33,000 formerly S-palmitoylated (S-acylated) peptides from the mouse brain membrane preparations. This highlighted tissue-specific S-palmitoylation events in mice organs, including synaptic signaling in brain, ion channels and transporters in kidney, and certain metabolic processes like bile secretion in liver. We identified S-palmitoylation of many enzymes involved in regulation of other PTMs, including kinases and phosphatases, strongly suggesting PTM crosstalk between S-palmitoylation and other PTMs. There was also extensive S-palmitoylation on zDHHC enzymes, which could serve as a reservoir for palmitoyl moieties for substrate S-palmitoylation. Several of the identified S-palmitoylated proteins were validated as being S-palmitoylated using a protein membrane-buffer partitioning method. A comparison between the SDC-ACE and the widely used RAC methods revealed that the former significantly outperformed the latter approach.

This refined SDC-ACE approach enabled the novel identification of several significantly regulated S-palmitoylated peptides in obesity-linked diabetic mouse brain, including S-palmitoylation belonging to proteins involved in insulin and leptin signaling pathways. This implicates a previously unrealized protein S-palmitoylation PTM associated with common metabolic disorders.

## Experimental Procedures

### Materials

Sodium deoxycholate (SDC), N-Ethylmaleimide (NEM), Iodoacetamide, and Dithiothreitol (DTT) were obtained from Sigma Aldrich. Trifluoroacetic acid (TFA), Hydroxylamine (HA) and Formic acid (FA) was obtained from Merck, Germany. Tris(2-carboxyethyl)phosphine (TCEP) was obtained from Thermo Fisher Scientific, Germany.

### Experimental Design and Rationale

This study describes a novel method, and we have employed an experimental design to ensure robust proteomic analysis while balancing ethical and practical considerations. To illustrate the application of the method using standard tissue samples and for method comparison, we analyzed two biological replicates per condition, which showed high reproducibility in our PCA plots, supporting the reliability of our findings. The comparison between different de-palmitoylating agents was performed with four technical replicates. The comparison of depalmitoylated peptides from the *db/db* and control mouse brains was performed using four biological replicates.

### Animals and Harvesting of Tissues

Wild-type (WT) C57BL/6J and C57BL/6J^db/db^ mice were originally purchased from The Jackson Laboratory and housed on a 12-h light/dark cycle with free access to standard mouse food and water. Mice were sacrificed between 14 to 16 weeks of age and brains were isolated immediately after and stored at −80 °C until further processing. Animal care, use, and experimental protocols were all approved by the Institutional Animal Care and Use Committee (IACUC number ACUP 71797) of the University of Chicago (Chicago, IL, USA).

Mice of strain NMR1 were euthanized at age 1 to 3 months, and whole brains were excised rapidly, flushed in 0.1% saline, and snap frozen in liquid nitrogen. The various organs (liver, kidney, heart, brown fat and white fat) were dissected and snap frozen in liquid nitrogen. All organs were stored at −80 degrees until use. All animal work was performed at the University of Southern Denmark animal facility (https://www.sdu.dk/en/om-sdu/institutter-centre/biolab_biomedicinsk_laboratorium) in accordance with the Animal Welfare Body (IACUC) at the University of Southern Denmark (License number 2021-15-0201-01054).

### Synthetic Peptides

The three tryptic synthetic peptides used for this study (See Table 1) contain a single free cysteine (highlighted in bold in the table below) and no serine and threonine residues, eliminating the possibility of S-palmitoylation of these residues using the palmitoylation method. All other cysteines were carbamidomethylated. The C-terminal arginines were all heavily labeled with Arg-10 (^13^C6,^15^N4). The synthetic peptides were obtained as a gift from Prof. Phillip Robinson, Children’s Medical Research Institute.

**Table 1:** Synthetic peptides used for developing the SDC-ACE method

ID	Sequence
Peptide A	VAIHCHAGLGR
Peptide B	EEPGC^a^C^a^IAVHCVAGLGR
Peptide C	EEPGC^a^C^a^VAVHCVAGLGR

a Denote carbamidomethylated cysteines.

### Generation of S-Palmitoylated Synthetic Peptides

Synthetic S-palmitoylated peptides were generated essentially as described in (27). Synthetic peptides were S-palmitoylated by dissolving the dried peptides in 10 μl 100% TFA and subsequently adding 1 μl palmitoyl chloride (Sigma Aldrich, Steinheim, Germany). The solution was incubated for 10 min at room temperature (RT). After incubation, the solution was diluted to 5% TFA and 20% Acetonitrile (ACN) and centrifuged at 14,000g for 10 min. S-palmitoylated peptides were purified by mixing the solution with Oligo R1 material (Poros, Perseptive Biosystems, Framingham, USA) for 10 min at RT. After incubation, the beads were washed with 20% ACN, 0.1% TFA and the S-palmitoylated peptides were eluted with 60% ACN.

### MALDI MS of S-Palmitoylated Synthetic Peptides

Small aliquots of the S-palmitoylated and non-palmitoylated synthetic peptides (0.5 μl) were mixed with 0.5 μl 0.1% TFA and 0.5 μl α-Cyano-4-hydroxycinnamic acid (4-HCCA) on a stainless steel MALDI target (Bruker), and the mixture was allowed to dry at RT. The peptides were analyzed using a Bruker Ultraflex (Bruker Daltonics) in positive reflector ion-mode. An average of 4000 laser shots were used for each peptide to obtain a MALDI MS spectrum. For the mixture of S-palmitoylated synthetic peptides and tryptic peptides from lactoglobulin, the same procedure was used, except for the solution containing the wash of the SDC pellet. Here, the supernatant from the wash was concentrated on a Poros Oligo R2 microcolumn and the peptides were eluted directly onto the MALDI MS target using the MALDI MS matrix solution.

### Comparing De-Palmitoylation of Synthetic S-Palmitoylated Peptides Using HA, DTT, and TCEP

**Fig. 1:**
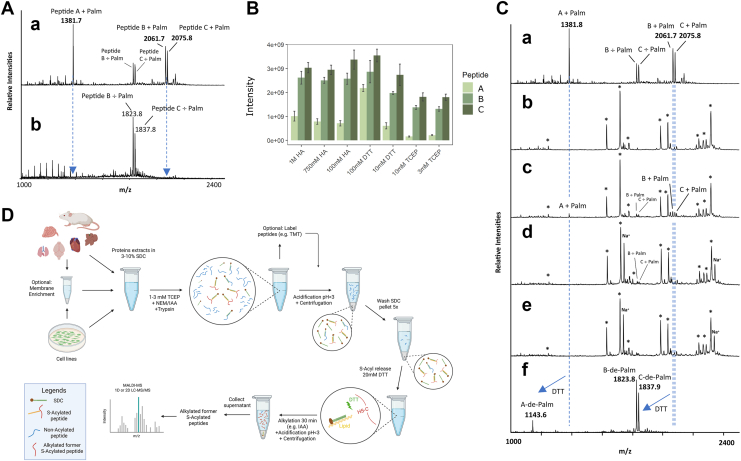
**Development of the SDC-ACE method for enrichment of S-palmitoylated peptides.***A*, (*a*) MALDI MS analysis of synthetic S-palmitoylated peptides (*b*) MALDI MS analysis of supernatant after SDC precipitation. *B*, validation of the most efficient reducing reagent for releasing S-palmitoylation from the synthetic S-palmitoylated peptides. DTT was comparably more efficient at releasing S-palmitoylation compared to HA and TCEP. *C*, specificity of the SDC-ACE method demonstrated by mixing synthetic S-palmitoylated peptides (*a*) with lactoglobulin derived peptides (*b*). The mix of peptides (*c*) shows both lactoglobulin peptides (∗) and synthetic Palm/÷Palm peptides. S-palmitoylated peptides are retained in the SDC pellet as shown from precipitation and washing supernatants (*d*-*e*). *f*, efficient release of de-palmitoylated peptides from the SDC fraction using DTT. *D*, Schematic representation of the SDC-ACE workflow using SDC acid precipitation for efficient S-palmitoylated peptide enrichment. Schematic representation was created with BioRender.com.

For comparing the de-palmitoylation efficiency of HA, DTT, and TCEP, S-palmitoylated synthetic peptides were mixed and diluted in 50 mM Tris containing 1% SDC, pH: 7.4. The pool of peptide solution was split into new tubes with equal volumes. Samples were then treated with HA, DTT, or TCEP at concentrations given in Figure 1*B* and incubated at RT for 1 h. Following incubation, SDC was precipitated by adding 100% FA to a final concentration of 2% and the samples were centrifuged at 14,000 *g* for 10 min to pellet the SDC.

Released de-palmitoylated peptides were then purified using Poros Oligo R3 reversed-phase (RP) microcolumns, by loading the acidified solution in equal volume to an equilibrated p200 stage tip containing C18 3M material disk (Fisher Scientific) and Oligo R3 material (Poros, Perseptive Biosystems, Framingham, US). Microcolumns were washed with 40 μl 0.1% TFA, and peptides were eluted with 40 μl 60% ACN in 0.1% TFA. Eluted samples were dried by lyophilization before LC-MS/MS.

### Label-Free Quantification of De-Palmitoylated Synthetic Peptides Using LC-MS/MS

The enriched de-palmitoylated samples were analyzed with a nano-Easy liquid Chromatography instrument (Thermo Fisher Scientific) coupled to a Q-Exactive HF mass spectrometer (Thermo Fisher Scientific). Dried samples were resuspended in 0.1% FA and loaded onto a 2 cm 100 ID pre-column containing C18 material (Reprosil 3 μm, Dr Maisch, Ammerbuch-Entringen) and eluted directly onto an analytical column with a gradient of 0% to 34% buffer B (90% ACN in 0.1% FA) over 23 min. All LC-MS/MS runs were performed with an analytical column of 18 cm × 75 μm inner diameter fused silica packed with C18 Reprosil 3 μm material (Dr Maisch, Ammerbuch-Entringen). Mass spectrometry was performed using full MS scan with a resolution of 120,000 and a target value of 3 × 10^6^. For MS/MS maximum injection time was 100 ms, and scan range 300 to 1300 m/z. Synthetic peptides were quantified based on the extracted ion chromatogram (XIC) intensity levels from raw data files.

### Enrichment of De-Palmitoylated Peptides From Mouse Brain Membrane Preparation

A brain from a C57BL/6J mouse (day 21 old) was homogenized using a Dounce homogenizer in 2 ml ice-cold 100 mM Sodium Carbonate (Na_2_CO_3_) for isolation of membrane proteins (28). After homogenization, the solution was subjected to probe sonication for 3 × 20 s at 60% amplitude on ice, using a Q125 Sonicator (Qsonia). The sample was then incubated at 4 °C with rotation for 1 h and, after, subjected to ultracentrifugation at 120.000*g* for 60 min at 4 °C. The supernatant was transferred to another tube and stored at −20 °C. The pellet containing membrane proteins, membrane associated proteins and larger protein complexes were washed with 50 mM HEPES, pH 8. It was then solubilized by sonication for 2 × 60 s in 500 μl 50 mM HEPES, pH 8, containing 10% SDC and subsequently placed at 110 °C for 5 min for complete inactivation of any enzymic activity. After this heat denaturation, the solution was diluted to 5% SDC and the sample was centrifuged at 20,000*g* for 20 min at RT. The supernatant was transferred to a low binding Eppendorf tube (Sorensen Bioscience Inc. 1,7 ml) and the protein concentration was measured using an Implen NanoPhotometer N60 (Implen).

A total of 2 mg of the membrane-enriched protein fraction was subjected to reduction of disulfide bridges using 3 mM TCEP for 30 min at RT and the free cysteines were subsequently alkylated using 20 mM NEM. After alkylation, the proteins were digested using Endoproteinase Lys-C (0.04AU) for 2 h at 37 °C. Then, trypsin (homemade modified trypsin (29) (5% w/w)) was added, and the sample was left overnight at 37 °C. Afterwards, FA was added to a final concentration of 2% (w/v) and the sample centrifuged at 20,000*g* for 20 min at RT to pellet the SDC. The supernatant was transferred to another low-binding Eppendorf tube, and the pellet was washed 3 times using 20% ACN, 50 mM HEPES and 1 M potassium chloride (KCL). Briefly, the pellet was solubilized by sonication (1 × 20 s at 40% amplitude), and NaOH was added to increase the pH to >8.0 for complete solubilization of the SDC. The SDC was then precipitated using FA (final of 2% (v/v)) and centrifuged at 20,000*g* for 20 min. After the three washes, the SDC pellet was washed two times further with H_2_O buffered to pH 8 by NaOH to solubilize the SDC in a similar way as described above. All washes were dried by lyophilization. After the last wash, the SDC pellet was solubilized in 600 μl 50 mM HEPES and adjusted to pH 8 using NaOH. An aliquot (20 μl) was transferred to another low-binding Eppendorf tube, and to the remaining solution, DTT was added to a final concentration of 20 mM. An aliquot (20 μl) was transferred to a low-binding Eppendorf tube. All three samples were then incubated at RT for 60 min. Afterwards, FA was added to the two 20 μl aliquots to a final of 2% (v/v) and the samples were centrifuged at 20,000*g* for 20 min to pellet the SDC. These two samples were subsequently analyzed by LC-MS/MS for indication of the efficiency of releasing de-palmitoylated peptides from SDC. The third sample, containing the remaining 560 μl de-palmitoylated sample, was alkylated using iodoacetamide (40 mM) for 45 min at RT in darkness. After this alkylation, FA was added to a final of 2% (v/v), and the sample was centrifuged at 20,000 g for 20 min to pellet the SDC. After centrifugation, the supernatant was subjected to Poros Oligo R3 RP microcolumn desalting, and the peptides were subsequently lyophilized for high pH RP separation. The procedure was repeated twice from the membrane extraction, to give a duplicate experiment.

### Comparison Between Acyl-RAC and the SDC-ACE Methods

Mouse brain membrane preparations from four brains from NMR1mice were prepared using the sodium carbonate extraction as described above. After ultracentrifugation, the pellets were combined by probe sonication in 1 ml 500 mM HEPES, pH 8.5 containing 3 mM TCEP and 20 mM NEM and incubated at RT for 1 h in darkness. Then, the sample was again ultracentrifuged at 120,000*g* for 1 h. The supernatant was discarded. The pellet was solubilized in 2 ml 1% SDS, 100 mM HEPES, pH 7.5 by probe sonication. Protein concentration was measured by amino acid composition analysis (30). Membrane proteins were aliquoted into four vials (each containing 2 mg protein) for two control (H_2_O) and two 1M HA treatments using the RAC method for enrichment of palmitoylated proteins (23). Briefly, H_2_O was added to the two control vials and 2M HA was added to the HA treated vials to reach 1M HA. The pH was adjusted to 7.5 and the samples were incubated for 1 h at RT. After this incubation, 200 μl slurry of S3 high-capacity acyl-RAC capture beads (NANOCS) were added to each vial and the samples then incubated with rotation for 2 h at RT. The S3 beads were pelleted by gentle centrifugation in a table centrifuge for 10 s, and the supernatant was removed. The pellets were washed three times with 500 μl 100 mM HEPES, pH 7.5 and twice with 500 μl H_2_O using MobiSpinColumns “F” with 10 μm filters (MoBiTec, #M105010S). After washing, the beads were suspended in 300 μl 1% SDC 100 mM HEPES, pH 8.5, and subsequently 2 μg Trypsin added. The samples were left overnight for digestion at 37 °C. After digestion, the peptides were recovered by centrifugation through the filter and the S3 beads were washed three times with 500 μl H_2_O. After washing, the S3 beads were resolubilized in 300 μl 100 mM HEPES, pH 8.5 containing 20 mM DTT and incubated at 37 °C for 1 h. After incubation, the released peptides were recovered by centrifugation and subsequently alkylated by 45 mM Iodoacetamide for 30 min at RT darkness. After this alkylation, the solution was acidified by FA to 1% (v/v) and the peptides desalted using Oligo R3 RP microcolumns. Peptides were eluted from the R3 material with 60% Acetonitrile and lyophilized. The dried peptides were dissolved in 0.1% FA (v/v) and analyzed by LC-MS/MS using the Orbitrap Astral MS instrument. For comparison with SDC-ACE, the SDC-ACE protocol described above was applied to 500 μg membrane protein starting material in triplicates. Briefly, a total of 3 mg of the solubilized membrane protein in 1% SDS, 100 mM HEPES, pH 7.5 was applied to 10 KDa spin filters (Millipore) and the buffer exchanged to 5% SDC 100 mM HEPES, pH 8.5. After washing, the proteins on the spin filters were digested with Trypsin (3% (w/w)) overnight at 37 °C. Then, another 1% (w/w) Trypsin was added, and the samples further incubated at 37 °C for 1 h. The peptide solution was recovered into two low binding Eppendorf tubes and the SDC was precipitated with FA. After washing the SDC pellet five times (as described above), the two SDC pellets were solubilized in 2 × 1 ml 100 mM HEPES, combined and adjusted to pH 8.5. The solution was divided into six tubes and diluted once with 100 mM HEPES, pH 8.5. To three of the tubes 13.3 μl 1M DTT was added to achieve a final 20 mM DTT. To the remaining three tubes 13.3 μl H_2_O was added as a control. The samples were incubated at 37 °C for 1 h, then iodoacetamide added to a total of 45 mM to each sample and further incubated at RT for 30 min in darkness. After this alkylation, the samples were acidified with 2% FA (v/v) and centrifuged for 20 min at 20,000*g* at RT to pellet SDC. The supernatants were transferred to low-binding Eppendorf tubes and peptides desalted using Oligo R3 RP microcolumns. Peptides were eluted from the R3 material with 60% Acetonitrile and lyophilized. The dried peptides were dissolved in 0.1% FA (v/v) and then analyzed by LC-MS/MS using the Orbitrap Astral MS instrument.

### Validation of Protein S-Palmitoylation by Protein Membrane-Buffer Partitioning

Mouse brain membrane preparations from brains from NMR1 mice were prepared using the sodium carbonate extraction as described above. After ultracentrifugation, the pellets were combined then sonicated in 1 ml 500 mM HEPES, pH 8.5. followed by ultracentrifugation at 120,000g for 1 h at 4 °C. The supernatant was discarded. The pellet was resolubilized by probe sonication to a slurry in 1.5 ml 100 mM HEPES, pH 7.5 and an aliquot was taken for measuring protein concentration using amino acid composition analysis (30). A total of 100 μg membrane protein was taken out into 12 ultracentrifuge Eppendorf tubes, and each was diluted to 200 μl membrane slurry using 100 mM HEPES, pH 7.5. Four replicates were treated with H_2_O, four with 1M HA and four with 50 mM DTT. The four tubes for DTT treatment were adjusted to pH 8 using NaOH. After incubation at RT for 1 h, the tubes were ultracentrifuged at 120.000*g* for 1 h at 4 °C. Then, the top 100 μl of the supernatant was carefully transferred to low-binding Eppendorf tubes, and the reduced proteins were washed using a 10-KDa filter (Millipore) two times using 1% SDC in 100 mM HEPES, pH 8.5. The washed proteins were then reduced with 5 mM DTT for 30 min at RT followed by alkylation with 10 mM iodoacetamide in darkness for 30 min at RT. After alkylation, the proteins were subjected to trypsination overnight at 37 °C. After trypsination, the SDC was precipitated using acid precipitation and centrifugation as above. The supernatant was collected and dried by lyophilization. The supernatants were resolubilized in 20 μl 0.1% FA (v/v), and 5 μl was analyzed by LC-MSMS using an Orbitrap Astral mass spectrometer for label-free quantitation.

### Analysis of De-Palmitoylated Peptides From Mouse Tissue Using TMTpro Quantitation

Membrane and membrane-associated proteins were isolated from various mouse tissues (2 replicates for each tissue) using bead beating in 1 ml 100 mM ice-cold Na_2_CO_3_. The bead beating was done using a FastPrep-24 instrument (MP Biomedicals) with a total of 25 ceramic beads per sample in a 2 ml Eppendorf tube. After bead beating, the sample was transferred to a 1.5 ml Eppendorf tube and sonicated for 2 × 20 s at 60% amplitude on ice using a Q125 probe-sonicator (QSONICA). The supernatant was collected and transferred to a Microtube WX 1.5 ml ultracentrifuge tube (Thermo Scientific Cat. No. 314352H01), incubated for 60 min a 4 °C with rotation, then subsequently centrifuged at 120,000*g* for 60 min at 4 °C using a HITACHI micro ultracentrifuge CS 150NX. The supernatant was removed, and the pellet was then washed with 100 mM HEPES, pH 8. The washed membrane protein pellet was solubilized in 200 μl 10% SDC in 50 mM HEPES, pH 8.5 by probe-sonication (2 × 20 s at 40% amplitude using the Q125 probe-sonicator). After sonication, the sample was diluted to 5% SDC (w/v) using 50 mM HEPES, pH 8.5 and then the protein concentration was measured using an Implen NanoPhotometer N60 (Implen).

A total of 100 μg of protein from each tissue (six tissues with two replicates from each tissue) and a mixture of proteins from all tissues, was taken and subjected to reduction using 3 mM TCEP and alkylation using 10 mM NEM. After alkylation Endoproteinase Lys-C (0.04AU) was added for 1 h at 37 °C and subsequently trypsin was added (5% (w/v)) and incubated overnight at 37 °C. After proteolysis, TMTpro was added to each solution for labeling as following; Kidney (TMT126/127N); Heart (TMT127C/128N); Brain (TMT128C/129N); Liver (TMT129C/130N); brown adipose tissue (BAT) (TMT130C/131N); white adipose tissue (WAT) (TMT131C/132N); Mixture (134N). After TMT labeling the samples were mixed and the SDC was precipitated using 2% FA (v/v) and centrifugation at 14,000*g* for 10 min at RT. The SDC pellet was washed and the de-palmitoylated peptides were isolated as described above. The enriched de-palmitoylated peptides were lyophilized prior to high pH RP separation.

### Analysis of De-Palmitoylated Peptides From Mouse WT and *Db/db* Brain Using TMTpro Quantitation

Proteins from the mouse brains were isolated using 200 μl 5% SDC in 50 mM HEPES, pH 8.5 by probe-sonication (3 × 20 min at 60% amplitude (Q125 probe-sonicator). After sonication, the solutions were centrifuged at 14,000*g* for 20 min at 4 °C. The supernatant was transferred to a low-binding Eppendorf tube and the protein concentration measured using an Implen NanoPhotometer N60 (Implen, Germany). A total of 100 μg of protein from four replicates of WT and db/db brains was transferred to new tubes and proteins were reduced, alkylated, and digested with Endoprotease Lys-C and trypsin as described above. The four replicates of WT and *db/db* brains were labeled with TMTpro (WT (126, 127N, 128N and 129N); *db/db* (130N, 131N, 132N and 133N)). After labeling, the samples were pooled, and the de-palmitoylated peptides were isolated as described above. The enriched de-palmitoylated peptides were lyophilized prior to high pH RP separation.

### High pH RP Separation

The high pH RP separation was performed essentially as described (31). The de-palmitoylated peptides from the two replicates of the mouse brain membrane preparation were separated into 12 concatenated fractions directly into a microtiter plate. The de-palmitoylated peptides from the mouse tissue TMT experiment were separated into 20 concatenated fractions and the de-palmitoylated peptides from the *db/db* mouse brain TMT experiment were separated into 12 concatenated fractions. All the fractions were lyophilized prior to LC-MS/MS.

### Liquid Chromatography Tandem Mass Spectrometry (LC-MS/MS)

#### Mouse Brain Membrane De-Palmitoylated Peptides

The 12 concatenated fractions from each high pH separation (replicates) were resolubilized in 5 μl 0.1% FA and 3 μl was loaded onto a nano-Easy LC (Thermo Fisher Scientific) coupled to an Orbitrap Eclipse mass spectrometer (Thermo Fisher Scientific). Samples were loaded onto a 20 cm 100 ID column containing C18 material (Reprosil 1.9 μm, Dr Maisch, Ammerbuch-Entringen). Replicates 1 and 2 were analyzed using a 60 to 90-min LC gradient. Briefly, the peptides were eluted with a gradient 2 to 25% buffer B (90% ACN in 0.1% FA) over 50 to 70 min, 25 to 40% buffer B over 10 to 20 min and finally 40 to 95% buffer B in 1 min. Peptides were selected for 3 s cycle time for MS/MS using higher energy collision dissociation (HCD) with normalized collision energy (NCE) setting as 32 to 33, resolution of 15 to 30.000 FWHM, Automatic Gain Control (AGC) target value of 300% and maximum injection time of 100 msec.

#### Comparison of RAC and SDC-ACE

Solubilized peptides were loaded onto a PepMap Neo Trap RP cartridge using a Vanquish Neo UHPLC System (ThermoFisher Scientific). These peptides were eluted from the precolumn onto an EasySpray 50 cm 75 ID analytical column (ThermoFisher Scientific, ES903) and eluted directly into an Orbitrap Astral Mass spectrometer (ThermoFisher Scientific) using a gradient from 2% to 45% buffer B (95% Acetonitrile in 0.1% FA) for 60 min. MS1 mass range m/z 350 to 1500 was acquired in the Orbitrap with an AGC target of 250% at a resolution of 240.000 FWHM with a cycle time of 0.6 s. Data-dependent acquisition (DDA) MS2 was performed in the Astral analyzer with an isolation window of 0.7 Da, HCD fragmentation of 30%, AGC target value of 300% and a filling time of 10 msec.

#### Membrane-Buffer Partitioning Validation Experiment

Solubilized peptides from control, HA and DTT treatment of the membrane slurry were loaded directly onto a Aurora Elite 15 × 75 C18 UHPLC column (IonOpticks, Melbourne, Australia) using a Vanquish Neo UHPLC System (ThermoFisher Scientific). These peptides were eluted from the column directly into an Orbitrap Astral Mass spectrometer (ThermoFisher Scientific) using a gradient from 2 to 45% buffer B (95% Acetonitrile in 0.1% FA) for 30 min. MS1 mass range m/z 350 to 1500 was acquired in the Orbitrap with an AGC target of 500% at a resolution of 240.000 FWHM with a cycle time of 0.6 s. DDA MS2 was performed in the Astral analyzer with an isolation window of 1 Da, HCD fragmentation of 30%, AGC target value of 200% and a filling time of 5 msec.

#### Mouse Tissue TMTpro Experiment

The 20 concatenated fractions from each high pH separation were resolubilized in 3.5 μl 0.1% FA (v/v) and 3 μl was loaded onto a 20 cm 100 ID column containing C18 material (Reprosil 1.9 μm, Dr Maisch, Ammerbuch-Entringen) on a nano-Easy LC (Thermo Fisher Scientific) coupled to an Orbitrap Exploris 480 mass spectrometer (Thermo Fisher Scientific). The peptides were eluted using a 90 min gradient as described above. Peptides were selected for 3 s cycle time for MS/MS using isolation window of 0.7 Da, HCD with NCE 33, a resolution of 45.000 FWHM, AGC target value of 300% and maximum injection time of 100 msec.

#### Mouse WT and Db/db TMTpro Experiment

The 12 concatenated fractions from each high pH separation were resolubilized in 3.5 μl 0.1% FA and 3 μl loaded onto a 20 cm 100 ID column containing C18 material (Reprosil 1.9 μm, Dr Maisch, Ammerbuch-Entringen) on a nano-Easy LC (Thermo Fisher Scientific) coupled to an Orbitrap Exploris 480 mass spectrometer (Thermo Fisher Scientific). These peptides were eluted using a 120 min gradient (2–30% buffer B (90% ACN in 0.1% FA) over 100 min, 30–50% buffer B over 20 min and finally 50–95% buffer B in 1 min). Peptides were selected for 3 s cycle-time for MS/MS using isolation window of 0.7 Da, HCD with NCE 33, a resolution of 45.000 FWHM, AGC target value of 300% and maximum injection time of 100 msec.

### Peptide Identification and Quantitation

All raw files from each replicate for the mouse brain S-palmitoylome mapping were searched in Proteome Discoverer (PD) 2.5.0.400 (Thermo Fisher Scientific) using both the SEQUEST HT (EMBL_mouse.fasta database (21.816 sequences), and Mascot (Uniprot database of *Mus musculus,* 25.775 sequences) search algorithms. Search parameters were as follows: cleavage specificity trypsin/P; precursor mass tolerance of 10 ppm, and fragment mass tolerance of 0.05 Da. Variable modifications: Methionine oxidation, N-terminal Acetylation, N-terminal Met-loss, N-terminal Met loss + Acetyl, NEM(C) and carbamidomethyl (C). For all searches two missed cleavages were used.

For the Orbitrap Astral MS data (i.e. comparison between the RAC and SDC-ACE and the membrane treatments) label-free quantitation was performed using the PD software with the SEQUEST HT search algorithm. Briefly, the four raw data files from the two replicates from the RAC-control and RAC-HA experiments were searched in PD using the EMBL mouse proteome database (21.816 sequences) with the following search parameters: cleavage specificity trypsin (full); precursor mass tolerance of 10 ppm, and fragment mass tolerance of 0.03 Da. Variable modifications: Oxidation (M), NEM(C) and carbamidomethyl (C). For quantitation, the Minora Feature Detector node was used to extract XICs for each peptide, and the Precursor Ion Quantifier node was used to perform the quantitation based on the XICs. The build in *t* test in PD was used for statistical measurements to identify potential de-palmitoylated peptides using the RAC method.

For the SDC-ACE comparison with 500 μg starting material, the database search was performed with the same settings but without the quantitation as the controls contained below 100 cysteine-containing peptides in each replicate.

For the validation experiment the 12 raw data files (4xControl, 4xHA and 4xDTT) were uploaded to PD and searched in SEQUEST HT using the EMBL mouse proteome database (21.816 sequences) with the following search parameters: cleavage specificity trypsin (full); precursor mass tolerance of 10 ppm, and fragment mass tolerance of 0.05 Da. Fixed modification: carbamidomethyl (C). For quantitation, the Minora Feature Detector node was used to extract XICs for each peptide and the Precursor Ion Quantifier node was used to perform the quantitation based on the XICs. The build in *t* test in PD was used for statistical measurements to calculate *p*-values for the reported quantitation ratios.

For the TMTpro experiments, the raw files were searched in PD 2.5.0.400 using both the SEQUEST HT and Mascot search algorithms as described above. Search parameters were as follows: cleavage specificity trypsin/P; precursor mass tolerance of 10 ppm, and fragment mass tolerance of 0.05 Da. Variable modifications: NEM(C) and carbamidomethyl (C). Fixed modifications: TMTpro (K and N-term).

For all database searches, the percolator software in PD was used for filtering for false discovery rate (FDR) of <1% for peptides. All data sets from PD were exported to Excel (Microsoft) for further processing.

### Bioinformatics

Venn diagrams were created using the Venn.diagram package in R. All datasets containing unique sites from the SwissPalm database were downloaded 31-05-2023 (Release 4, 2022-09-03). Gene Ontology (GO) and Kyoto Encyclopedia of Genes and Genomes (KEGG) analyses of identified de-palmitoylation sites in the mouse brain samples were performed using the Cytoscape StringApp (ver. 2.0.0) in Cytoscape (Ver.3.10.1) (32, 33). Prior to GO/KEGG analysis, data were clustered using Markov clustering algorithm (MCL) clustering with the Cytoscape ClusterMaker2 (ver. 2.3.2) app (34). GO/KEGG analysis was performed separately on each cluster against the entire dataset as background. Redundant terms were filtered out and only GO terms (cellular compartment, molecular function and biological processes) and KEGG pathways were considered. To space nodes in the string map for better visualization, we used the yfiles Layout Algorithm (ver. 1.1.2) organic layout function. For comparing and removing disulfide bonds, we used Uniprot disulfide annotation data (downloaded 12-02-2024) and filtered our data using an R script. Analysis of mouse organ tissues were performed in R with kmeans clustering and using both R and Cytoscape StringApp for GO/KEGG analysis and visualization. Normalized abundance values from PD of each replicate sample were divided with a mixed replicate sample (MIX) followed by log_2_ transformation and scaling. Log_2_ z-scores were used for heatmap and kmeans clustering. For StringApp GO/KEGG analysis, identified S-palmitoylated peptides assigned to different clusters were filtered for only those identified peptides having at least one replicate across all conditions with a log_2_ z-score value above 2, to minimize number of nodes in each visualization. Statistical tests of *db/db* mouse brain palmitoylome were performed using PolySTest (35). The *db/db* mouse brain data were log_2_ transformed prior to statistical test and S-palmitoylated peptides with an FDR-value <0.05 were considered statistically significant. Volcano plot was generated using R. For Cytoscape StringApp analysis of db/db mouse brain samples, we performed enrichment analysis on clusters of proteins from all statistically significant S-palmitoylated peptides identified with a genome-wide background. For clustering, we used MCL clustering. (36). Graphical structure of WDFY1 were created from icn3D accessed at National Center for Biotechnology Information (37).

## Results and Discussion

### Development of the SDC Acid Precipitation Enrichment (SDC-ACE) Method for S-Palmitoylation

**Figure S1:**
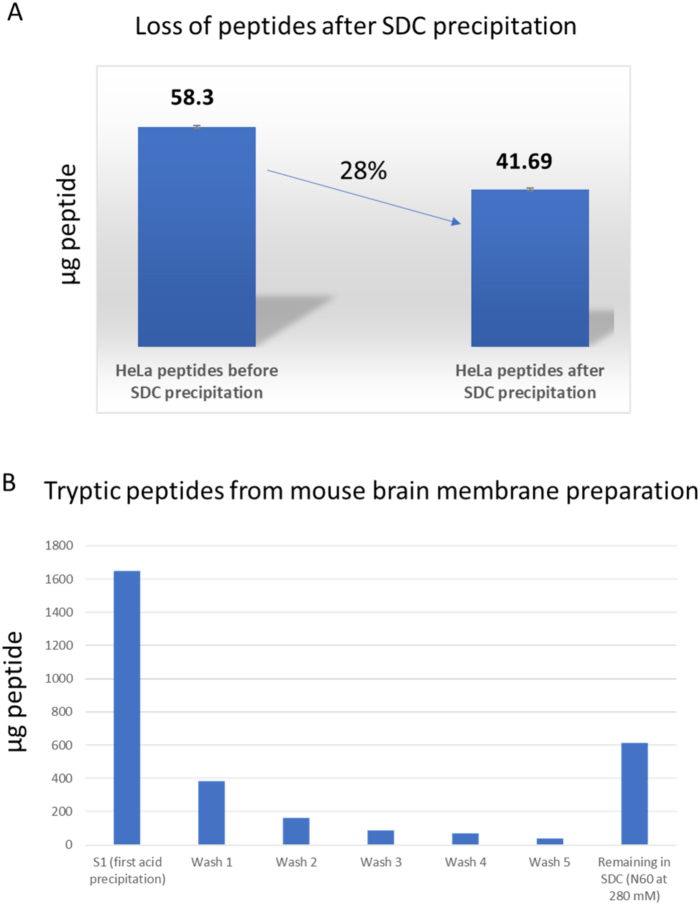


SDC is a frequently used detergent in proteomics for extracting proteins from cells and tissues, since most proteases, such as trypsin, retain activity in SDC (38). SDC is easily removed using either phase transfer extraction with a water-immiscible organic solvent, like ethyl acetate (39), or acid precipitation followed by centrifugation (40). Whereas phase transfer extraction often results in contamination due to the top ethyl acetate phase, the acid precipitation is fast and easy. However, yield from acid precipitation is lower resulting in a loss of peptides to the SDC pellet. This is illustrated by the tryptic digestion of an aliquot of proteins extracted from HeLa cells using 1% SDC. The content of peptides was measured using Amino Acid Composition analysis before and after acid precipitation and centrifugation (Supplemental Fig. S1*A*). The acid precipitation resulted in almost 30% loss of peptide amount to the SDC pellet. But, when washing the SDC pellet by re-solubilization in pH 8 to 8.5 buffer followed by acid precipitation, more peptides can be recovered from the SDC pellet (41). This is illustrated here by the tryptic digestion of proteins extracted from a mouse brain membrane preparation using 5% SDC (w/v) (Supplemental Fig. S1*B*). Here, the recovery of primarily increasingly hydrophobic peptides could be detected in repeated washes of the SDC pellet (Supplemental Fig. S1*B*). This, together with the relatively large amount of remaining material in the SDC pellet, indicates that very hydrophobic peptides bind tightly to the precipitated SDC. We therefore hypothesized that lipid modified peptides are enriched in the SDC pellet.

**Figure S2:**
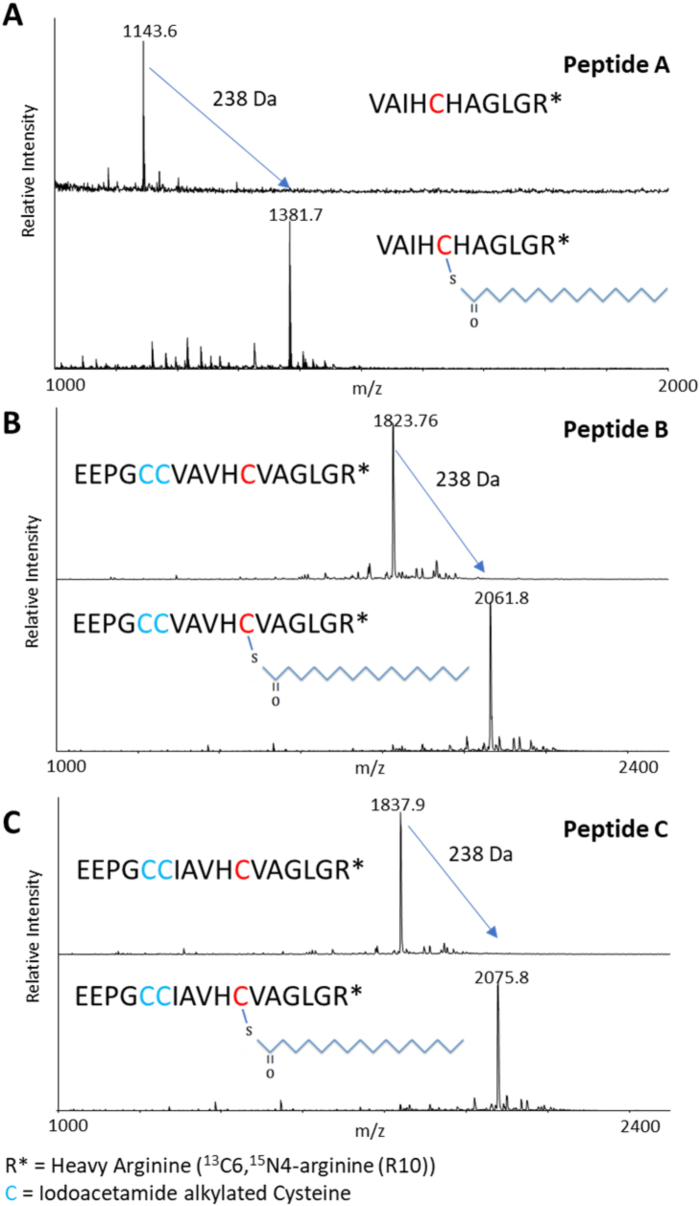


To investigate this hypothesis, three synthetic S-palmitoylated peptides (peptide sequence A, B and C) were generated using TFA and palmitoyl chloride (27) (Supplemental Fig. S2, *A*–*C*). To test if S-palmitoylated peptides co-precipitate with the SDC after acidification and centrifugation, these three S-palmitoylated peptides (A-, B-, and C-Palm) were combined and analyzed by MALDI-MS (Fig. 1*A*a). SDC was added to the mixture to a final concentration of 1% (w/v) followed by acidification and centrifugation. MALDI-MS analysis of the supernatant revealed their complete removal (Fig. 1*A*b), indicating efficient enrichment of the S-palmitoylated peptides with SDC acidic precipitation. Unfortunately, analysis of the intact S-palmitoylated peptides from the SDC pellet was not possible by MS analysis due to the high amount of SDC present.

**Figure S3:**
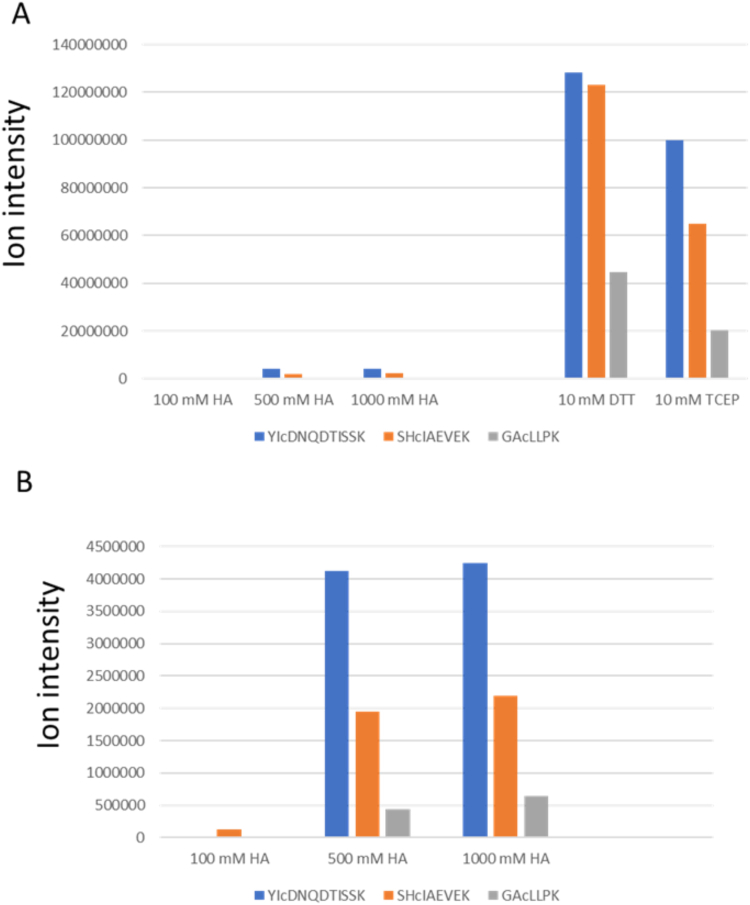


The three peptides were subsequently incubated with common reagents used for reduction of reversibly modified cysteines (e.g., disulfide bonds) and thioesters; Tris(2-carboxyethyl)phosphine (TCEP), DTT and HA, to test their efficiency in releasing the S-palmitoylation. Traditionally, HA has been used for decades for the selective release of palmitoyl groups from cysteines (20), albeit with very little evidence for its selectivity. Efficient and selective enrichment of formerly de-palmitoylated peptides using the ABE and Acyl-RAC methods require that HA is quite selective in releasing the palmitoyl group as these methods subsequently link biotin (or any other tag) to newly generated free SH group followed by enrichment and very sensitive LC-MS/MS analysis. However, the ability of HA to reduce abundant disulfide bonds has, to our knowledge, never been assessed. We therefore tested the HA specificity on bovine serum albumin, where the free cysteines were blocked with N-ethylmaleimide (NEM) prior to precipitation of the protein. After tryptic digestion, we assessed the reduction of the disulfide bonds left in the protein using DTT, TCEP and various concentrations of HA. From the result of the experiment (Supplemental Fig. S3), it is evident that higher concentrations of HA, as the ones used in most ABE and Acyl-RAC experiments, leads to a reduction of a small amount of disulfide bonds in BSA. This results in subsequent contamination of formerly S-palmitoylated peptides with abundant disulfide-bonded cysteines in previous studies that have used more conventional methods for identifying de-palmitoylated peptides.

**Figure S4:**
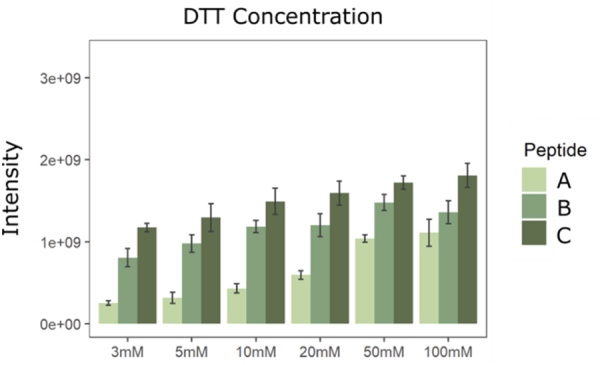


To test the reducing reagents’ ability to reduce the thioester bond, the synthetic S-palmitoylated peptides were incubated with different concentrations of HA, DTT and TCEP for 60 min and then de-palmitoylated peptides were quantified using LC-MS/MS (Fig. 1*B*). DTT was the most efficient of the three reducing reagents and we therefore chose 20 mM DTT as the optimum DTT concentration for release of de-palmitoylation peptides from the SDC pellet (Supplemental Fig. S4). This concentration shows the most efficient de-palmitoylation of the standard palmitoylated peptides used here and does not require high concentration of subsequent alkylating reagents. For the reduction of disulfide bonds in our method we chose 1 to 3 mM TCEP as this resulted in minimal release of S-palmitoylation.

The specificity of the SDC enrichment of the S-palmitoylated peptides in the background of peptides derived from bovine lactoglobulin was examined by MALDI-MS analysis (Fig. 1*C*a-c). After adding SDC to a final concentration of 1% (w/v) followed by acidic precipitation and centrifugation, the supernatant (S1) was analyzed and signals originating from the S-palmitoylated peptides were absent, indicating their precipitation with the SDC (Fig. 1*C*d). Washing the precipitated SDC pellet only recovered remnant peptides from lactoglobulin, as expected (Fig. 1*C*e). De-palmitoylation using 20 mM DTT on the re-solubilized SDC pellet resulted in the release of all three de-palmitoylated peptides (Fig. 1*C*f), illustrating relatively high selectivity of the method.

### Large-Scale SDC-ACE Strategy

We designed the optimized SDC-ACE strategy for enriching formerly S-palmitoylated peptides using SDC acid precipitation to be applied to cells and tissues (Fig. 1*D*). Proteins are first extracted using 3 to 10% SDC (optimal analysis is obtained using a crude membrane preparation (42)), reduced using 1 to 3 mM TCEP which is adequate for most applications and alkylated using 10 mM NEM. The proteins are then proteolyzed using trypsin or Lys-C/trypsin. After this proteolytic digestion, the peptide solution is subsequently acidified and SDC pelleted by centrifugation. The SDC pellet is subsequently washed 3 times with washing buffer and twice with H_2_O, all buffered to pH 8-8,5 using NaOH. Finally, reduction of S-palmitoylation is performed using 20 mM DTT in 50 mM HEPES, pH 8, and subsequent alkylation of de-palmitoylated cysteines using iodoacetamide. De-palmitoylated peptides are collected from the supernatant following SDC acid precipitation and centrifugation and they are subsequently analyzed by 1D or 2D LC-MS/MS after desalting. A small volume of the SDC sample without DTT served as a LC-MS/MS background control for the de-palmitoylation step. The SDC-ACE method can be combined with quantitative proteomics methods such as TMT and label free quantitation. However, using label free quantitation methods requires that this final workflow is optimized further to secure high reproducibility, which could be difficult with the extensive washes of the SDC pellet in the protocol (Fig. 1*D*). Here the TMT approach would be easier to implement.

### The Mouse Brain S-Palmitoylome

**Fig. 2:**
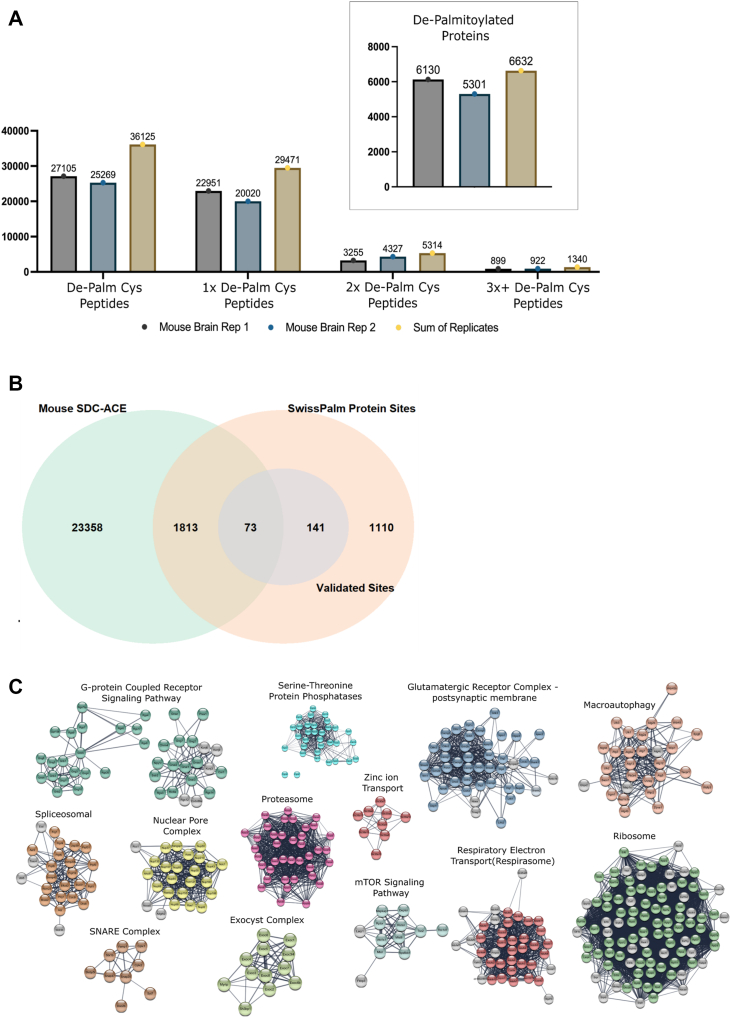
**The S-palmitoylation from mouse brains.***A*, the number of identified de-palmitoylated peptides and proteins in each of the two replicates and the sum of both. Peptides are separated based on the number of S-palmitoylation modification sites in a tryptic peptide, ranging from one to 3+. *B*, comparison of mouse brain S-palmitoylation sites identified by the SDC-ACE method with all unique S-palmitoylated sites, including experimental validated unique sites in the SwissPalm database. *C*, enrichment of GO and KEGG-pathway terms of the SDC-ACE enriched mouse brain S-palmitoylation proteins using the Cytoscape StringApp. Multiple protein complexes are illustrated.

**Figure S5:**
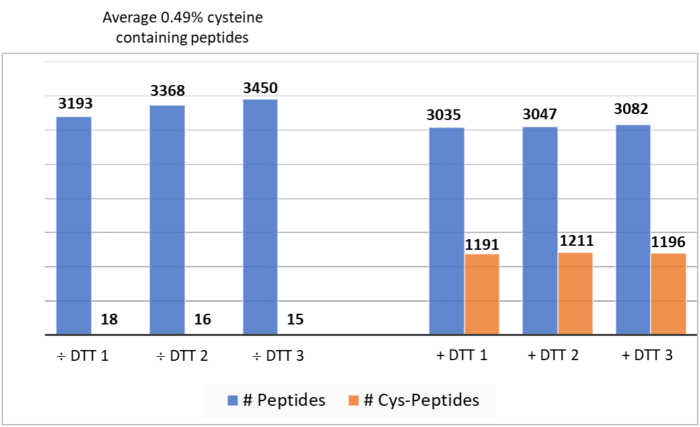


The SDC-ACE method was applied to a crude membrane preparation from mouse brain in replicates using a total of 2 mg protein as starting material per replicate, which is comparable with other studies assessing formerly S-palmitoylation sites in cells or tissues. The enriched de-palmitoylated peptides were separated into 12 concatenated fractions using high pH reversed-phase (RP) separation. From the two replicates a total of 36,125 unique cysteine-containing peptides (Supplementary file: MouseBrainPalmProteome table S1–S3) were identified from 6632 proteins (Fig. 2*A*). A small aliquot of the SDC pellet analyzed with and without DTT revealed a “false discovery rate” in isolation of cysteine-containing peptides estimated to be approx. 0.49% (Supplemental Fig. S5). A total of 81.6% of the de-palmitoylated peptides contained one cysteine, whereas 18.4% of the de-palmitoylated peptides contained two or more closely spaced cysteines, generating highly hydrophobic hubs on protein surfaces likely for interaction with lipid membranes, proteins, or other biomolecules (Fig. 2*A*). Comparing the identified mouse brain palmitoylated proteins with the list of known and candidate palmitoylated proteins (Group A, high spectral count support) identified by ABE/MudPIT in rat embryonic cortical neurons and rat synaptosomal membrane fractions in the study of Kang *et al*. (43), we identify 56 out of 71 of the known palmitoylated proteins in the study and 61 out of 113 of the identified candidate palmitoylated proteins in that study. The study conducted by Kang *et al* was performed using the ABE method where HA is used for “selective” reduction of S-palmitoylation. As shown in Supplemental Fig. S3, HA can result in the reduction of abundant disulfide bonds and the proteins that are not found in our experiment could well be due to the use of HA.

As also noted by Collins *et al* (24), we observed that some of the identified S-palmitoylation sites in our data mapped to cysteines annotated as situated in disulfide bonds (experimentally determined and predicted) in the Uniprot database. S-palmitoylation and disulfide bonds are not mutually exclusive and therefore both reversible cysteine modifications could occupy the same sites. We have previously observed that SDC is capable of co-precipitating proteins (data unpublished) and therefore larger disulfide-linked peptides could potentially co-pellet with the SDC. Therefore, to increase confidence in our list of de-palmitoylated peptides, we filtered out all identified peptides with cysteine sites annotated as situated in disulfide bonds according to the UniProt annotation (Supplementary file: MouseBrainPalmProteome table S5). This included multi-de-palmitoylated peptides even if only a single cysteine was annotated in the UniProt disulfide database. We found that 2794 sites from our mouse brain data mapped to cysteines annotated as disulfide bond forming cysteines in the UniProt database, corresponding to 9.9% of all identified sites (28,256 sites in total) in the mouse brain data. A total of 128 non-disulfide cysteine sites were filtered out, as they were located on multi-de-palmitoylated peptides with at least a single disulfide annotated cysteine. This result compares well to the 6.5% that was previously observed by Colins *et al*. (24). However, it is worth noting that S-palmitoylation has previously been reported to occur on cysteines involved in disulfide bond formation (44). Thus, these sites may very well be S-palmitoylated in our samples.

Our list of mouse brain de-palmitoylated peptides share 1886 of the 3137 unique S-palmitoylation sites from predictions, compiled palmitoyl-proteome and validation studies in the mouse SwissPalm database (45, 46) (60.12%), including 73 of the only 214 validated unique sites in the database (Fig. 2*B*). Sites from SwissPalm were filtered for only SwissProt curated proteins. Some of the validated sites were not identified due to the tryptic cleavage specificity generating less likely detectable peptides in the LC-MS/MS analysis and UniProt disulfide annotation. Considering the use of HA in previous studies for “selective” reduction, and the difficulty in predicting S-palmitoylation by bioinformatics tools, we assume that a significant number of false de-palmitoylation sites is present in the SwissPalm database, contributing to a reduced overlap between sites in our dataset.

Gene Ontology (GO) and Kyoto Encyclopedia of Genes and Genomes (KEGG) pathway analysis of proteins containing peptides with S-palmitoylation sites was highly associated with known larger protein complexes, such as the ribosome, spliceosome, proteosome, SNARE-complex, Exocyst complex, mTOR-complex 1, Complex I-V and ATPase Complexes (mitochondrial respiratory system, respirasome) (Fig. 2*C*). S-palmitoylation has previously been associated with some of these complexes (4, 45, 47). However, the present results strongly suggest that S-palmitoylation may be of much broader importance for formation, interaction and regulation of protein complexes via protein-protein interaction in the cell.

**Figure S6:**
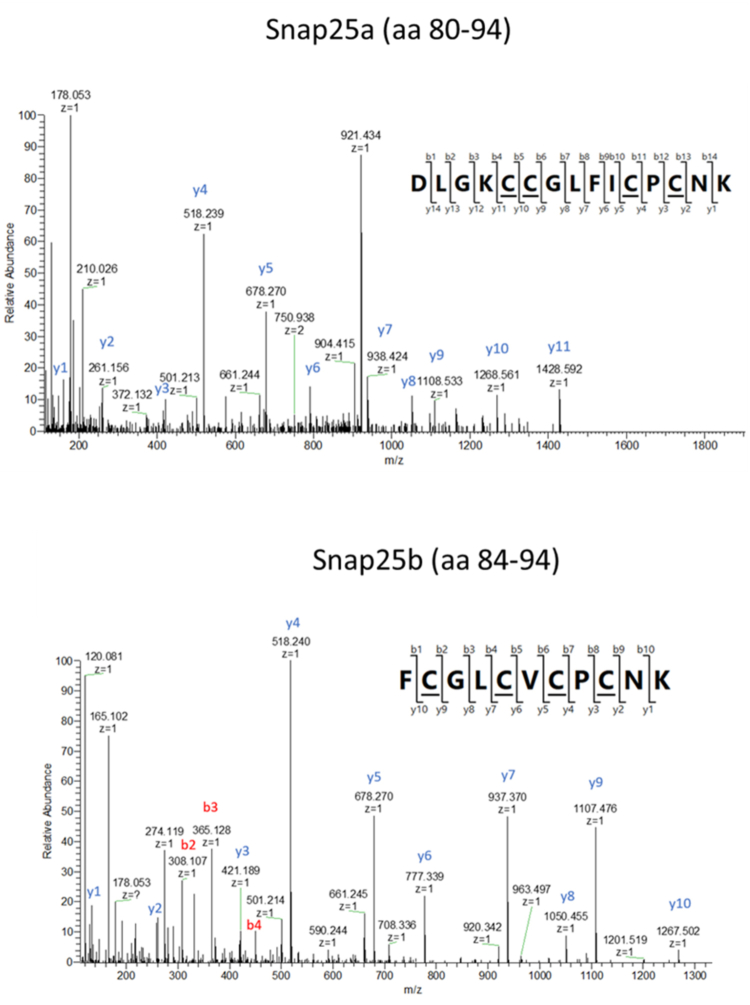


**Figure S7:**
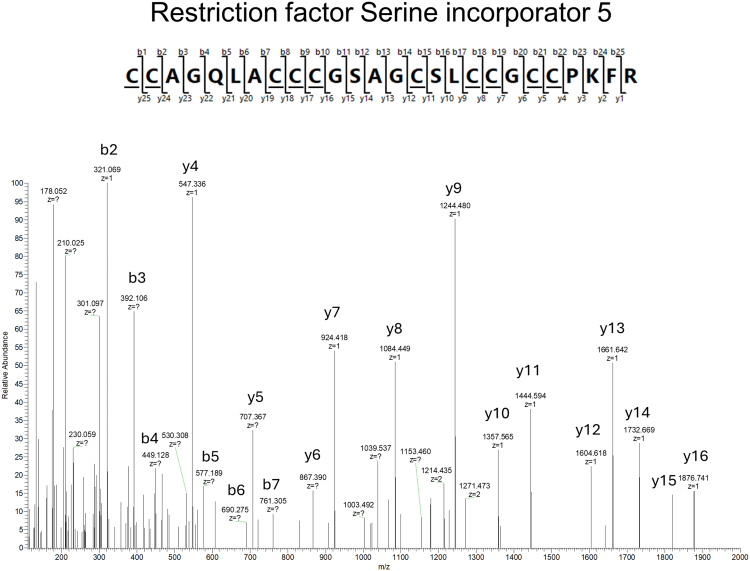


A total of 1340 peptides from 748 proteins, resulting in 1110 peptides from 647 proteins after disulfide correction contained three or more cysteines located in the same tryptic peptide sequence, indicating the presence of hydrophobic clusters in short regions in these proteins (Fig. 2*A*). In support of the approach, several well-known multi-S-palmitoylated proteins were identified, such as SNAP25a/b (annotated spectra for the de-palmitoylated peptides in Supplemental Fig. S6, *A* and *B*), that is part of the SNARE complex involved in synaptic vesicle exocytosis, as well as PSD-95, a postsynaptic scaffolding protein required for synaptic plasticity (1). A peptide from the N-terminal, extracellular part of Serine incorporator 5 (SERC5) (Uniprot: Q8BHJ6), an important protein in viral infection and neurological disease (48), was identified containing 10 de-palmitoylated sites in a single tryptic peptide (Supplemental Fig. S7). Furthermore, S-palmitoylation sites were identified on multiple enzymes involved in regulatory processes in the cells including E3 ubiquitin-protein ligases, protein kinases, protein phosphatases, acetyltransferases, deacetylases, GTPases, and surface receptors, strongly suggesting that this modification may be considerably more involved in regulatory processes in the cell than previously thought.

**Figure S8:**
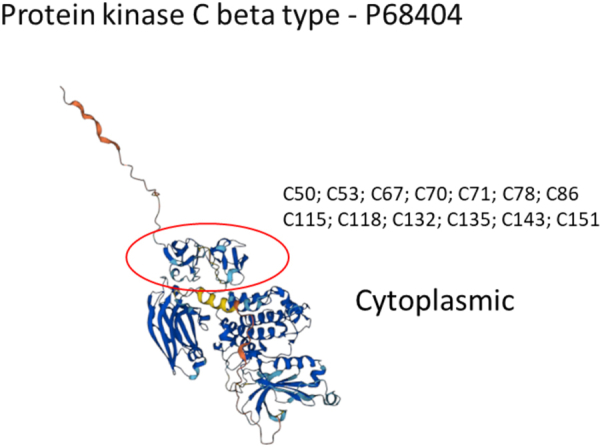


More than 350 kinases were identified in our dataset with potential S-palmitoylation sites, including small-molecule kinases and protein kinases. These include well-characterized important kinases in health and disease, such as ribosomal protein S6 kinases, Mitogen-activated protein kinases, Glycogen synthase kinase-3A/B, Cyclin-dependent kinase 5, Calcium/calmodulin-dependent protein kinases and various tyrosine kinases such as epidermal growth factor receptor, Tyrosine-protein kinase Fyn/Lyn/UFO/TYRO3/JAK1/JAK2/Fer/CSK/ALB2/FLT3 and many others. Some of these protein kinases were identified as heavily S-palmitoylated. For example, we identify a total of 21 de-palmitoylation sites in Protein Kinase C beta type, which is a kinase involved in several signaling pathways in the cell and is activated by calcium and diacylglycerol. This protein has two zinc finger domains (phorbol-ester/DAG-type 1 and 2) in the regions aa 36 to 86 and aa 101 to 151 (49). However, we identify the cysteines in these regions to be S-palmitoylated in our data set (Cys 50, 53, 67, 70, 71, 78, 86, 115, 118, 132, 135, 143 and 151). The presence of these peptides in the SDC pellet due to conjugation with zinc is highly unlikely as these peptides were released by DTT reduction, which will not interfere in zinc conjugated peptides. To support this observation, we washed a fraction of a SDC pellet containing peptides from mouse brain membrane preparation with 10 mM EDTA in 50 mM HEPES, pH 8.5 for 30 min at 37 °C followed by acid precipitation. Subsequent LC-MS/MS analysis of the released peptides did not result in the identification of cysteine-containing peptides (data not shown). The N-terminal domain of Protein Kinase C beta type, illustrated by a red circle in the 3D structure (see Supplemental Fig. S8), contains several loops and disordered regions and heavy S-palmitoylation of this region could drive the localization of this kinase to an intracellular membrane compartment, including the plasma membrane. Protein kinase C isoforms have previously been shown to be recruited to the plasma membrane after glucose-stimulation of pancreatic β-cells, but this translocation mechanism is unknown (50). This could illustrate the consequence of dynamic palmitoylation as being involved in directing intracellular localization and keeping signal transduction localized.

Phosphatases, responsible for dephosphorylation, were also found to be S-palmitoylated in our mouse brain dataset (e.g., Fig. 2*C*), including the abundant brain Ca^2+^-dependent phosphoprotein phosphatase calcineurin. Interestingly, several dual specificity protein phosphatases and tyrosine-protein phosphatases were found to be potentially modified by S-palmitoylation on the cysteine residue in the active site, in the HC(X)5R motif (51, 52), a site that must be free for the enzyme to be active. This suggests that S-palmitoylation may shield dephosphorylation activity for these phosphatases in the cell or drive subcellular localization.

The S-palmitoylation of multiple enzymes responsible for the dynamics of reversible PTMs in cells strongly suggests a crosstalk between S-palmitoylation and other PTMs. Such a PTM crosstalk has previously been suggested for protein phosphorylation (53, 54), S-nitrosylation (55, 56, 57) and ubiquitination (58, 59).

### Palmitoylation of the Mouse Nuclear Pore Complex in the Brain

The nuclear pore complex (NPC) is a critical structure that functions as a gateway between the nucleus and the cytoplasm in cells. It is composed of multiple proteins known as nucleoporins, which together form a large, octagonal complex (60). This complex regulates the selective transport of molecules, such as RNA and proteins, in and out of the nucleus. In mice, as in other eukaryotes, the NPC is essential for maintaining cellular function and gene expression (60). Structurally, it features a central channel surrounded by a ring of proteins, facilitating the bidirectional flow of cellular materials. Many of the proteins in the NPC are closely associated with the nuclear membrane (60).

**Fig. 3:**
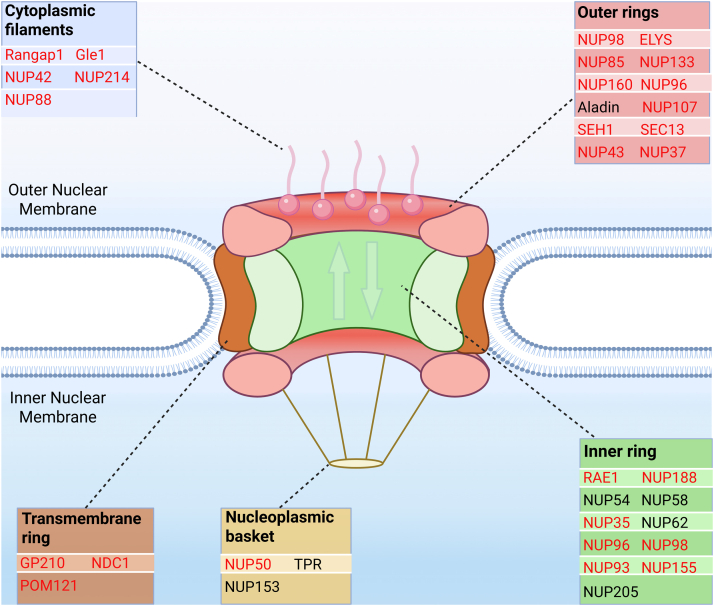
**S-palmitoylated components of the nuclear pore complex identified from the mouse brain.** Schematic illustration of the nuclear pore complex architecture that spans the nuclear envelope. Enriched de-palmitoylated peptides from the mouse brain could be identified from several proteins (highlighted in *red*) related to the nuclear pore complex. A total of 25 proteins known in the nuclear pore complex were identified as S-palmitoylated. These included proteins from the inner and outer rings and proteins situated in the nucleoplasmic basket, transmembrane ring and the cytoplasmic filaments. The figure was created with BioRender.com.

Among the proteins of the nuclear pore complex (60, 61), S-palmitoylation sites were identified on 25 in our study (Fig. 3 and Supplementary file: MouseBrainPalmProteome table S4)). These included proteins from the inner and outer rings and proteins situated in the nucleoplasmic basket, transmembrane ring, and the cytoplasmic filaments. This suggests that S-palmitoylation may be central to the structure, assembly, and/or dynamics of the nuclear protein complex and, therefore, important for the cytoplasmic-nuclear transportation of proteins and mRNA molecules.

### Palmitoylation of Palmitoyl-Transferases in the Mouse Brain

**Fig. 4:**
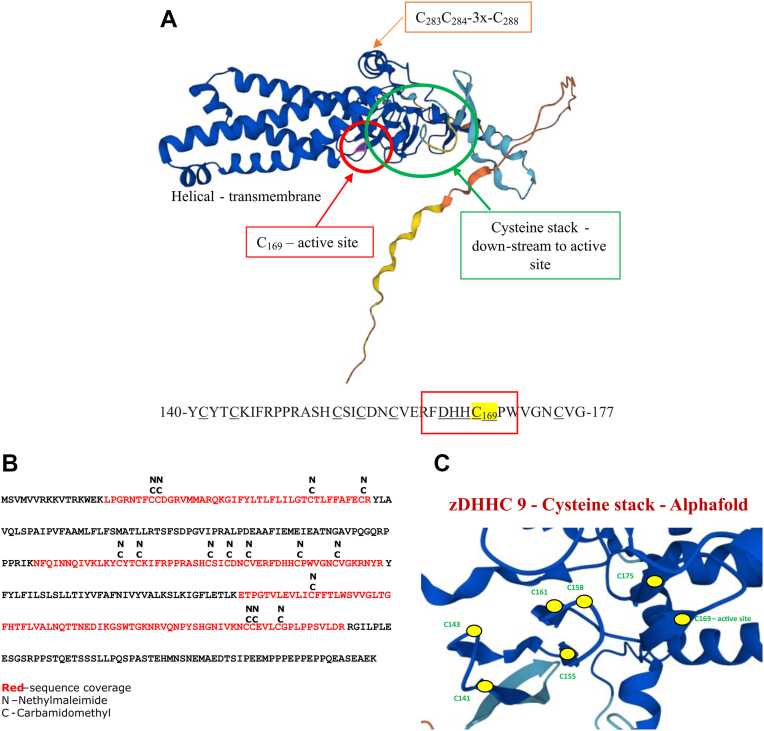
**3D structure and S-palmitoylation of zDHHC9.***A*, Alphafold 3D structure of zDHHC9 highlighting the cysteine stack (*green circle*), the active site (*red circle*) and C-terminal S-palmitoylation. *B*, Sequence coverage of zDHHC9 from Proteome Discoverer, highlighting de-palmitoylated and free cysteines (de-palmitoylated cysteines – carbamidomethyl (c) and free cysteines – ethylmaleimide (n)). *C*, Alphafold 3D structure illustration of the “Cysteine stack”, the active cysteine site and the upstream cysteine (*yellow dot* illustrate the individual cysteines).

As mentioned earlier, 24 zDHHC PATs exist in the mouse genome and several PATs are published to be S-palmitoylated by other PATs. A common feature of the zDHHC PATs is a transmembrane region and a cysteine rich domain (CRD-domain), that contain several downstream cysteines to the catalytic DHHC cysteine, illustrated with the 3D structure for zDHHC9 obtained from Alphafold (62, 63, 64) in Figure 4*A*. According to the 3D structures of all the zDHHC proteins, derived by Alphafold, the downstream cysteines are localized in sequential “stacks” close to the active site DHHC cysteine, as illustrated for zDHHC9 in Figure 4, *A*–*C*. Some of these cysteines have been suggested to participate in zinc binding as described for the zDHHC3 (15). Within the CRD amino acid sequence downstream of the proposed active DHHC site, several tryptic cleavages sites are located (K/R), and the presence of lipids in this area could compromise the tryptic cleavage specificity. Therefore, to investigate the S-palmitoylation of the CRD cysteines we searched the mouse brain de-palmitoylation data in a limited database containing only the zDHHC sequences using up to four missed cleavages in the database search criteria in Proteome Discoverer (PD). Since we blocked the free cysteines with NEM and labeled the formerly palmitoylated cysteines with iodoacetamide we used both modifications as variable modifications. The identified peptides were validated and filtered for 1% FDR using the percolator software (65) in PD. The database search result for zDHHC9 is shown in Figure 4*B*. We identified all the 24 zDHHCs in the mouse brain palmitoylome. The result of the database search for the other zDHHCs can be seen in the Supplementary File “zDHHC 3D structures”, where the sequence coverage from the database search is illustrated for each zDHHCs next to the 3D structures obtained from Alphafold. As evident from the peptide coverage, all the down-stream cysteines in all zDHHCs are potentially S-palmitoylated at some point in the mouse brain, but not fully S-palmitoylated, as illustrated by the differential presence of NEM on several cysteines. A cysteine located six amino acids upstream from the active site, conserved in all zDHHCs, was also found to be differentially S-palmitoylated in most of the zDHHCs in our data. As we cannot calculate any stoichiometry from our data, to comment on the level of S-palmitoylation on these cysteines would be too speculative.

Since previous research has shown that the cysteines within the CRD could be conjugated to zinc (15), it should be noted that washing the SDC pellet containing peptides from the mouse brain membrane preparation included the cation chelator EDTA. Subsequent LC-MS/MS analysis of the released peptides did not result in the identification of cysteine-containing peptides, such as the ones covering the CRD cysteines. This suggests that the CRD cysteines are not conjugated to zinc in the SDC pellet. However, this does not rule out that a fraction of these cysteines could be conjugated to zinc at some point in considering a dynamic characteristic of S-palmitoylation PTM in cells.

Nonetheless, these results strongly indicate that the conserved cysteines in the CRD and the upstream cysteine are sub-stoichiometric S-palmitoylated. The presence of S-palmitoylated cysteines in very close proximity to the active site cysteine (illustrated in Fig. 4*C*) could have several consequences. The up- and downstream cysteines to the active site are very closely situated in the 3D structure (Fig. 4*C* and Supplementary File “zDHHC 3D structures”) and therefore could serve as a reservoir of palmitate that can easily be transferred to the cysteine in the active site for S-palmitoylation of substrates. Alternatively, up- and downstream cysteines could be part of a larger conserved “active site pocket” where they all participate actively in the palmitoyl-transferase process, capable of transferring palmitate to the substrates. The latter is supported by the observation that mutations in the DHHC motif of yeast S-acyltransferases Swf1 and Pfa4 only partially abolish their activities (66).

### Comparison of SDC-ACE and Acyl-RAC S-Palmitoylation Enrichment

**Figure S9:**
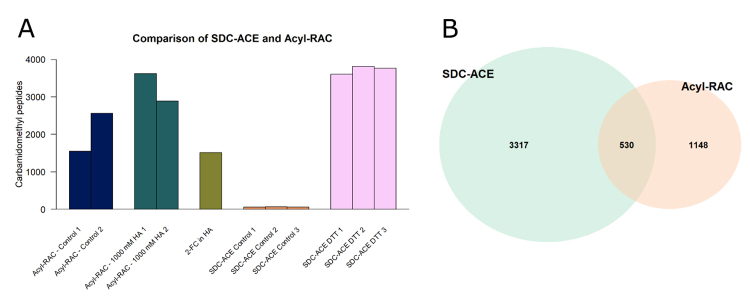


Acyl-RAC and ABE S-palmitoylation enrichment are the most common techniques used for enhancing S-palmitoylation analysis. But SDC-ACE offers a faster and easier enrichment procedure than Acyl-RAC and ABE by eliminating multiple chloroform-methanol precipitations and use of reagents such as thiopropyl Sepharose beads or biotin/streptavidin beads. Evaluating the performance differences between SDC-ACE and these methods may present differences in sensitivity, specificity and throughput, which could influence the selection of these methods for future studies. We compared our SDC-ACE method with the Acyl-RAC approach (23) for enriching S-palmitoylated proteins from mouse brain, using single-shot proteomics without high-pH RP pre-fractionation, as described for the entire mouse brain palmitoylome above. We performed Acyl-RAC on duplicates with HA and no HA treatment (control) from a total of 2 mg enriched crude membrane protein samples per replicate (as described above), and at the same time, we performed SDC-ACE in triplicates with or without (control) DTT from membrane protein samples containing a total of 500 μg protein per replicate. Enriched samples were analyzed by LC-MS/MS using an Orbitrap Astral mass spectrometer. From the Acyl-RAC samples, we identified 3622 and 2887 cysteine-containing peptides in the 1M HA-treated samples, and 1550 and 2562 cysteine-containing peptides in the control samples (Supplemental Fig. S9*A*). By performing quantitation using label-free XIC quantitation, we identified a total of 1516 cysteine-containing peptides that were identified with a 2-fold increase between the HA and control that were present in both replicates for each condition (Supplemental Fig. S9*A*). These were considered potential de-palmitoylated peptides as these criteria were used in previous RAC/ABE studies. For the SDC-ACE method, we identified more than 3600 potential de-palmitoylated peptides in each replicate of DTT-treated samples, resulting in more than 5300 unique potential de-palmitoylated peptides (Supplemental Fig. S9*A*). From the control triplicate experiments without DTT, we identified 60 to 69 cysteine-containing peptides in each replicate. The SDC-ACE method was applied to only 500 μg starting material in each replicate, whereas the RAC method was applied to 2 mg starting material in each replicate. The number of potential de-palmitoylated peptides is in line with other studies that frequently use>2 mg starting material for the RAC method (23, 24).

We identified 3847 potential unique de-palmitoylation sites using the SDC-ACE method and 1678 potential unique de-palmitoylation sites in the Acyl-RAC method (Supplemental Fig. S9*B*). Comparing the sites between the Acyl-RAC and SDC-ACE methods, there was an overlap of 530 sites (Supplemental Fig. S9*B*). Previous attempts to compare the ABE, Acyl-RAC and metabolic labelling approaches have revealed many unique protein identifications between the methods but with low overlap, which is likely due to methodological differences (67, 68). Similarly, we see a relatively low overlap in identified potential S-palmitoylation sites between SDC-ACE and Acyl-RAC. This could be because Acyl-RAC uses 1 M HA for reduction, which we have shown to result in a reduction of abundant disulfide bonds (Supplemental Fig. S3). However, we identify several well-known S-palmitoylated proteins and sites common in the Acyl-RAC and SDC-ACE methods, including, e.g. S-palmitoylation at site C328 and C329 in zDHHC6 (16), C88 and C90 in SNAP-25, and C109, C139, C141, C201, C220 and C228 in Myelin proteolipid protein.

### Validation of Lipidation of Selected Proteins by Membrane-Buffer Partitioning

Site-directed mutagenesis has been used to validate S-palmitoylation sites in certain proteins. However, the multifunctional nature of cysteine residues, which can be utilized by a variety of PTMs (including disulfide bonding, S-nitrosylation, S-glutathionylation, S-sulfenylation, S-sulfhydration, as well as S-palmitoylation) makes interpreting mutations challenging. Thus, utilizing this mutagenesis approach for confirming cysteine palmitoylation in proteins is limited and inconclusive.

**Fig. 5:**
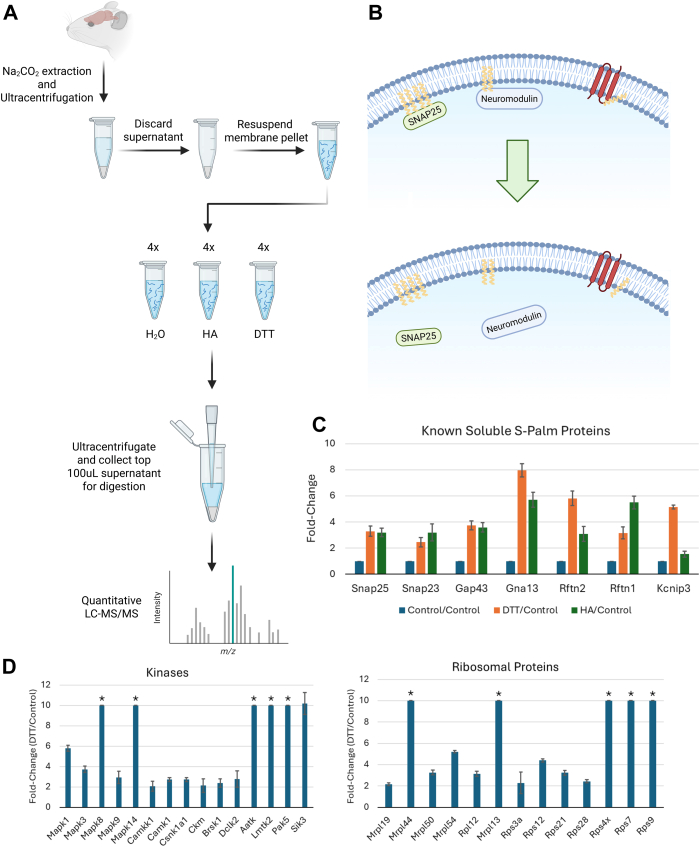
**Validation of S-palmitoylation by membrane-buffer partitioning.***A*, schematic representation of the validation study design. Mouse brains were subjected to membrane protein extraction by sodium carbonate treatment and ultracentrifugation. Solubilized membrane proteins were divided into 12 aliquots and treated with either H_2_O, HA or DTT followed by ultracentrifugation again and collection of top 100 μl supernatant for subsequent quantitative proteomics. Figure was created with BioRender.com. *B*, Cartoon illustration of the validation study design, illustrating the release of membrane-associated palmitoylated peptides into the supernatant upon DTT/HA treatment, leaving transmembrane proteins (*red*) remaining in the membrane fraction. Figure was created with BioRender.com. *C*, identified known soluble palmitoylated proteins in our validation study. *D*, kinases and ribosomal proteins identified as potential palmitoylated proteins in our validation study. The symbol ∗ marks proteins not identified in any control replicates, but in all 4 DTT replicates.

Instead, we developed an alternative approach using purified mouse brain membranes and larger protein complexes isolated by ultracentrifugation, combined with reduction, second ultracentrifugation, and quantitative LC-MS/MS analysis, an approach we termed protein membrane-buffer partitioning. We treated four replicates of a mouse brain membrane slurry in 100 mM HEPES buffer (100 μg each) with H_2_O, 1M HA, or 50 mM DTT for 1 h at RT. Post-treatment, the samples were ultracentrifuged at 120,000*g* for 1 h to pellet membrane material and larger protein complexes, and the top 100 μl of the supernatant was collected, trypsinized, and analyzed by quantitative LC-MS/MS (See Fig. 5*A* + B).

The results indicated significant increases in non-transmembrane proteins in the supernatant after reduction and ultracentrifugation, suggesting that these proteins were released from the membrane or from larger protein complexes by chemical reduction. This can only be attributed to the removal of a lipid attached to a cysteine. Supplementary file: MouseBrainPalmValidation Table S1 lists proteins that show a significant increase in abundance after reduction with HA or DTT compared to H_2_O treatment, including well-known palmitoylated proteins like SNAP23, SNAP25, Neuromodulin, and Raftlin-2, which were enriched more than 2-fold after both DTT and HA treatments (Fig. 5*C*). More proteins were significantly released from membranes after DTT treatment compared to HA treatment, which is in line with the results from the reduction of S-palmitoylated synthetic peptides, where DTT was more efficient than HA for reduction of S-palmitoylation from cysteines. No other modification on either cysteines or other amino acids can result in the release of soluble proteins from lipid membranes or larger protein complexes after DTT reduction.

A total of 477 proteins were found to be significantly enriched in the ultracentrifugation supernatant after DTT treatment compared with the H_2_O treatment (Supplementary file: MouseBrainPalmValidation Table S2), indicating S-palmitoylation of these. This number is relatively high considering the relatively low amount of starting material (100 μg per replicate) and the stringent criteria for quantitation, that the proteins had to be identified in all four replicates with at least two unique peptides to be considered for quantitation. More than 80% of the proteins (385 of 477) were found in our mouse brain S-palmitoylome (Supplementary file: MouseBrainPalmProteome Table S5). The remaining 20% could contain S-palmitoylated tryptic peptides that are not detectable by LC-MS/MS in our SDC-ACE approach due to size or other physiochemical properties. Among the “validated” lipidated (e.g., S-palmitoylated) proteins were members of the 26S proteasome (Psmd2, Psmd4, Psmd6 Psmd8 and Psmd10), the Ribosome (Rpl12, Mrpl13, Mrpl19, Mrpl44, Mrpl50, Rps3a, Rps4, Rps9, Rps12, Rps21 and Rps28), heterogeneous nuclear ribonucleoproteins (Hnrnpa3, Hnrnpd, Hnrnpdl, Hnrnph1 and Hnrnpm), the nuclear pore complex protein Nup98-Nup96, mitogen-activated protein kinases (1, 3, 8, and 9), protein phosphatase subunits (Ppp1r14a, Ppp2r2a, Ppp1r17 and Ppp3cb), Cullin-RING Ubiquitin Ligase complex proteins (Commd1, 2, 3, 5, 6, 8 and 9) (See Figs. 5*D* and S10). Several tyrosine kinases, such as Lyn or Fyn, were not identified here, probably due to the N-terminal myristylation of these kinases, which also has implications for associating them with membrane compartments, even after de-palmitoylation.

Notwithstanding, these findings support the efficacy of our methodology in identifying many S-palmitoylated proteins that is more straightforward and superior to a cysteine site-directed mutagenesis approach.

### Mouse Tissue-Specific S-Palmitoylation

The method was further applied to investigate S-palmitoylation characteristics of different mouse tissues using TMT-based quantitative proteomics in combination with SDC-ACE. After membrane enrichment, proteolysis, and TMT labeling, we enriched de-palmitoylated peptides from mouse brain, brown adipose tissue (BAT), white adipose tissue (WAT), heart, liver, and kidney. The enriched de-palmitoylated TMT labeled peptides were separated into 20 concatenated fractions using high pH RP separation, and each fraction was analyzed on LC-MS/MS. We identified a total of 18,575 de-palmitoylated peptides from 5926 proteins across all tissues (Supplementary file: MouseOrganTissuePalmProteome Table S1).

**Fig. 6:**
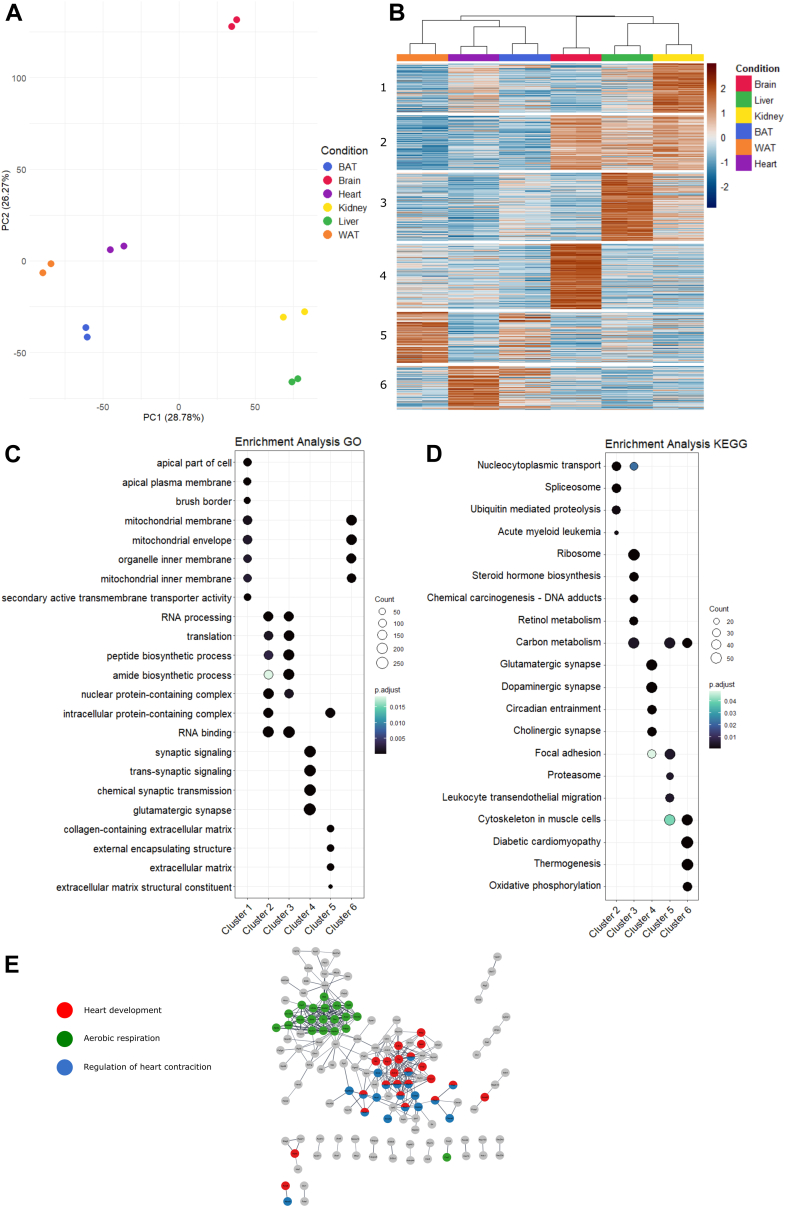
**Characterization of mouse tissue S-palmitoylomes.***A*, PCA analysis of mouse organ tissue palmitoylation. *B*, heatmap of kmeans clustered mouse organ tissues. Each organ includes two replicates. Scale bar refers to scaled log_2_ abundances for each replicate. Cluster numbers are listed on the left of the heatmap. *C + D*, GO/KEGG enrichment analysis of clustered mouse tissue. *E*, Cytoscape StringApp enrichment of GO/KEGG terms for mouse heart tissue following protein filtering for upregulated features with a log_2_ z-score value above two in at least one replicate across all conditions.

Removing cysteines potentially involved in disulfide bonds yielded 16,823 de-palmitoylated peptides (Supplementary file: MouseOrganTissuePalmProteome Table S2). As seen in the PCA analysis (Fig. 6*A*), the tissues are grouped into three separate clusters and the replicates cluster together. Clustering de-palmitoylated peptides revealed distinct patterns for most tissues (Fig. 6*B*), reflecting the physiological differences between these tissues. Duplicate samples from each tissue grouped together in the PCA plot, reassuring the specificity and reproducibility of our method. The mouse brain S-palmitoylome is different from the other tissues, most likely due to the particular functions of neurons as rapidly excitable cells, requiring abundant intracellular transport of vesicles and secretion of proteins and small molecules, processes known to be regulated by S-palmitoylation (69, 70, 71). Many of the unique S-palmitoylated proteins in the mouse brain are, as stated above, present in the nerve-terminals and axons where multiple transportation and secretion mechanisms exist. The kidney and liver group close to each other in the PCA plot, which could well be driven by S-palmitoylation of common metabolic pathways in these tissues.

GO-term and KEGG pathway enrichment of cluster four revealed terms mainly associated with the brain, such as synaptic signaling/transmission, agreeing with the increased enrichment of these de-palmitoylated peptides in the brain tissue (24) (Fig. 6, *C* and *D*). Similarly, in cluster 3, we enriched terms related to metabolism, such as amide/peptide biosynthetic processes and proteins involved in bile metabolism, showing a predominant association with the liver (See Supplemental Fig. S11), and we observed substantial S-palmitoylation on key proteins involved in small molecule transporters and ion channels in cluster 1, associated with the kidney (Supplemental Fig. S13). Cluster six represented S-palmitoylation abundant in the heart, revealed GO/KEGG pathways such as heart development, regulation of heart contraction and aerobic respiration (Fig. 6*E*). The impact of S-palmitoylation on cardiac physiology is not well-understood, with only a few known S-palmitoylated proteins with known roles in the heart (72). We identified several potential modification sites on Ryanodine receptor 2 (E9Q401) and S-palmitoylation of cysteine 1189 in the Voltage-dependent L-type calcium channel subunit Alpha 1C (Q01815), proteins involved in optimal myocardial function (72). In summary, these data support a role of S-palmitoylation in cellular processes across the entire body. The StringApp network GO/KEGG analyses for liver, brain and kidney can be seen in Supplemental Fig. S11–S13.

### Palmitoylation in Brain Tissue From Obesity-Linked Type 2 Diabetes (T2D) Mice

**Fig. 7:**
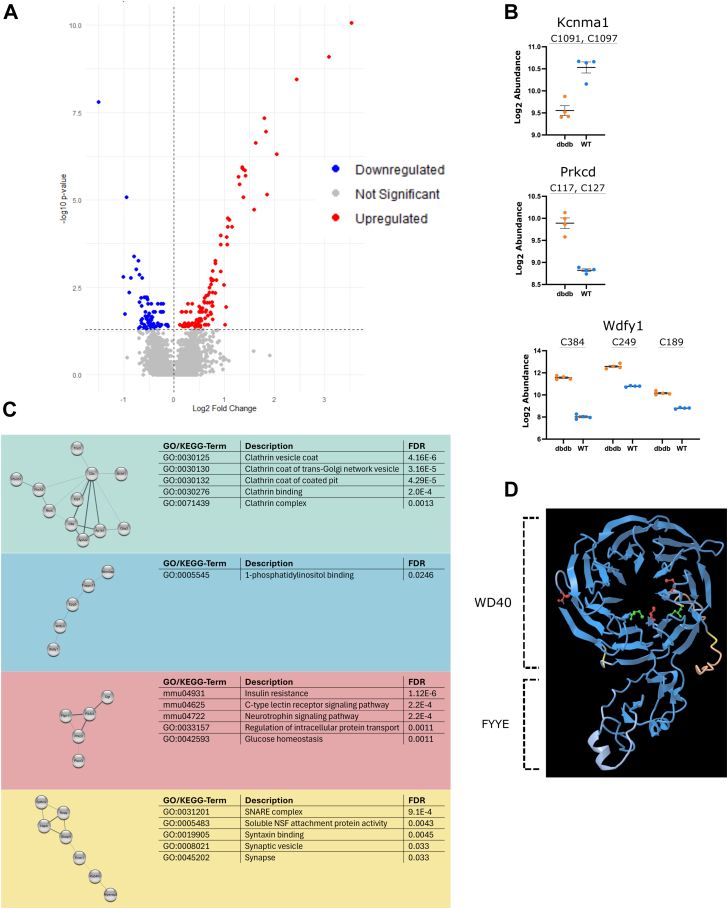
**Characterization of *db/db* mouse brain S-palmitoylome.***A*, Volcano plot of differentially regulated S-palmitoylated peptides enriched from Wild-type (WT) and *db/db* mouse brains. Significantly upregulated and downregulated S-palmitoylated peptides are shown in *red* and *blue*, respectively. Non-significantly regulated peptides are shown in *grey*. *B*, Scatter diagram of selected de-palmitoylated peptides identified in WT and *db/db* mice with relative log_2_-abundance. Numbers (e.g. C384) above scatter diagram correspond to S-palmitoylation site(s) identified on the respective peptide. Note that the peptides for Kcnma1 and Prkcd carries two palmitoylation sites. *C*, Cytoscape StringApp enrichment of protein clusters of significant (FDR <0.05) S-palmitoylated peptides identified in the *db/db* mouse brain based on PolySTest. The tables show the top five most significant GO/KEGG terms enriched for each cluster. Only one term was enriched for the cluster in *blue*. *D*, graphical illustration of WDFY1 showing the WD40 repeat domain and FYYE domain. Significantly regulated S-palmitoylation sites are illustrated and colorized in *red*. S-palmitoylation sites not significantly regulated, identified in our experiment, are shown and colorized in *green*.

We have previously profiled the proteome and phosphoproteome in relation to pancreatic β-cell adaptive restoration of insulin secretory capacity in obese diabetic *db/db* mouse islets, in which key molecular events in the secretory pathway reverted to normal upon euglycemic treatment (73). Considering that S-palmitoylation is quite likely related to trafficking/sorting events within the cell, we decided to apply our method to study the S-palmitoylome in brains from *db/db* mice. The *db/db* diabetic mouse model carries a mutation in the leptin receptor gene, inactivating the receptor and leading to hyperphagia, severe obesity, hyperglycemia and glucose intolerance (74). The model is widely applied for studying early phenotypes of obesity-linked T2D. The appetite-suppressing and weight-reducing effects of leptin is believed to be exerted primarily in the hypothalamus (75). Leptin receptor signals through a multitude of signaling pathways, including JAK2-STAT3, PI3K-AKT-mTOR, SHP2-ERK and more (75). Out of the 15,432 potential S-palmitoylated peptides identified in the analysis,198 were identified as significant S-palmitoylated peptides (FDR<0.05) (Fig. 7*A* and supplementary file: DiabeticMouseBrainPalmProteome table S1–S3). These sites were found in proteins such as Tyrosine-protein phosphatase non-receptor type 11 (Ptpn11, SHP-2), WD repeat and FYVE domain-containing protein 1 (WDFY1), Calcium-activated potassium channel subunit alpha-1 (Kcnma1), PI3K regulatory subunit beta (p85β) and protein kinase C delta type (PKC-δ) (Fig. 7*B*), proteins associated with signaling pathways of both insulin and leptin. Enrichment analysis of clustered significant S-palmitoylated peptides revealed GO/KEGG-terms such as insulin-resistance, SNARE complex, Clathrin vesicle coat and 1-phosphatidylinositol binding (Fig. 7*C*). WDFY1 belongs to a large family of WD40 repeat domain-containing proteins, one of the most common protein interaction domains in the human proteome (76). This domain consists of a central pore formed by circularly arranged beta-propeller folds, often acting as interaction scaffold proteins within multiprotein complexes (76). All identified S-palmitoylated cysteines in our analysis are localized to the WD40 domain (Fig. 7*D*). WDFY1 has been reported to localize to endosomes through its FYVE domain and act as an adaptor molecule in the Toll-like receptor (TLR) activation of NF-κB by TLR3/TLR4 signaling (77). However, the role of S-palmitoylation of WDFY1 in diabetic *db/db* mice is unknown.

Activation of hypothalamic PKC-δ by lipid modification has previously been reported to lower hepatic glucose production and regulate insulin-sensitivity by glucagon-like peptide 1 (GLP-1) (78, 79). In addition, PKC-δ^+^ neurons in the central amygdala have been implicated in regulating food intake. Interestingly, a recent paper found PKC-δ S-palmitoylated in hypothalamic microglia under high-fat diet-induced microglial neuroinflammation (80). Here, S-palmitoylation of PKC-δ was suggested to induce PKC-δ activation by facilitating T505 phosphorylation, leading to subsequent downstream signaling and contributing to microglial activation. Inhibiting PKC-δ S-palmitoylation using a derivative of artemisinin, artemether, suppressed neuroinflammation and alleviated hepatic lipid metabolism disorder in a mouse model (80). We found PKC-δ significantly upregulated on the doubly S-palmitoylated peptide carrying Cys117 and Cys127 in *db/db* mice (FDR<0.05, Log_2_-FC: 1.07), located just upstream of the two zinc finger domains phorbol-ester/DAG type 1 and 2 regions. The zinc fingers are located within the C1 domains, which are known as the binding site of novel PKCs to diacylglycerol (DAG) at the plasma membrane, a key step in the downstream signaling of novel PKCs (81). In addition, zDHHC5, a PAT mainly localized at the plasma membrane, were proposed as the PAT for PKC-δ S-palmitoylation (80). This indicates that PKC-δ is S-palmitoylated at the plasma membrane, potentially after recruitment and interaction with plasma membrane DAG, facilitating stable interaction with the plasma membrane or conformational changes for possible phosphorylation-mediated activation.

## Conclusion

The SDC-ACE method offers a highly efficient and more ‘user-friendly’ approach for identifying potential de-palmitoylation sites in biological samples. It integrates seamlessly with standard proteomics workflows that utilize SDC (sodium deoxycholate) during proteolysis.

By enabling effective enrichment of S-palmitoylated peptides before de-palmitoylation, SDC-ACE significantly reduces the risk of false identifications compared to conventional methods. This improvement is further strengthened by the omission of HA, which was shown to non-specifically reduce abundant disulfide bonds at high concentrations. Instead, selective reduction using DTT is employed after lipidated peptide enrichment, improving specificity and data quality.

A side-by-side comparison with the RAC method highlights the advantages of the SDC-ACE approach. SDC-ACE proved to be simpler, more sensitive, and capable of identifying significantly more potentially S-palmitoylated peptides, even when using a smaller amount of starting material. Furthermore, the subsequent exclusion of validated and putative disulfide-bonded cysteines from the final list of candidates S-palmitoylation sites enhances the stringency of the analysis. This conservative filtering step is particularly important, as some of these cysteines may be lipid-modified at sub-stoichiometric levels *in vivo*, potentially leading to misleading assignments if not properly accounted for. However, as for the other methods for targeting S-palmitoylation it has not been possible to analyze the S-palmitoylated peptides without reduction due to the tight binding to SDC which is contaminating the LC-MS/MS systems.

Applying our method to biological samples enabled us to characterize in depth the mouse brain palmitoylome, map tissue-specific S-palmitoylation between mouse organs and characterize the *db/db* mouse brain palmitoylome. It is evident that our novel SDC-ACE method has revealed a deep insight into S-palmitoylation of proteins, and further validation studies will be needed to gain deeper mechanistic insight into the function of palmitoylation of the proteins. Nonetheless, in the present study we highlight S-palmitoylation as an extensive PTM potentially involved in many regulative processes and associate S-palmitoylation with several tissue-specific pathways, implying S-palmitoylation could well be a critical PTM for cellular processes and signaling pathways in biological systems. Moreover, identifying S-palmitoylation on several important cellular enzymes responsible for regulation of other PTMs, including kinases and phosphatases, indicates a coordination of distinct PTMs that may specifically localize signal transduction activity to particular compartments in the cell. Finally, the observation that PKC-delta S-palmitoylation is significantly upregulated in the brain of obese diabetic mice, may indicate altered localized neuronal signaling pathways that potentially contribute to disease pathogenesis in obesity-linked T2D.

## Data Availability

The mass spectrometry proteomics data have been deposited to the ProteomeXchange Consortium via the PRIDE partner repository.

Project accession: PXD054682

Username: reviewer_pxd054682@ebi.ac.uk

Password: 9aqc2z7nTaFm

## Supplemental data

This article contains supplemental data.

## Conflict of interest

The authors declare the following financial interests/personal relationships which may be considered as potential competing interests: C. J. R. is an ex-employee of AstraZeneca and owns stock in the company. All other Authors declare no competing interests.

## Figures and Tables

**FIGURE∼1:**
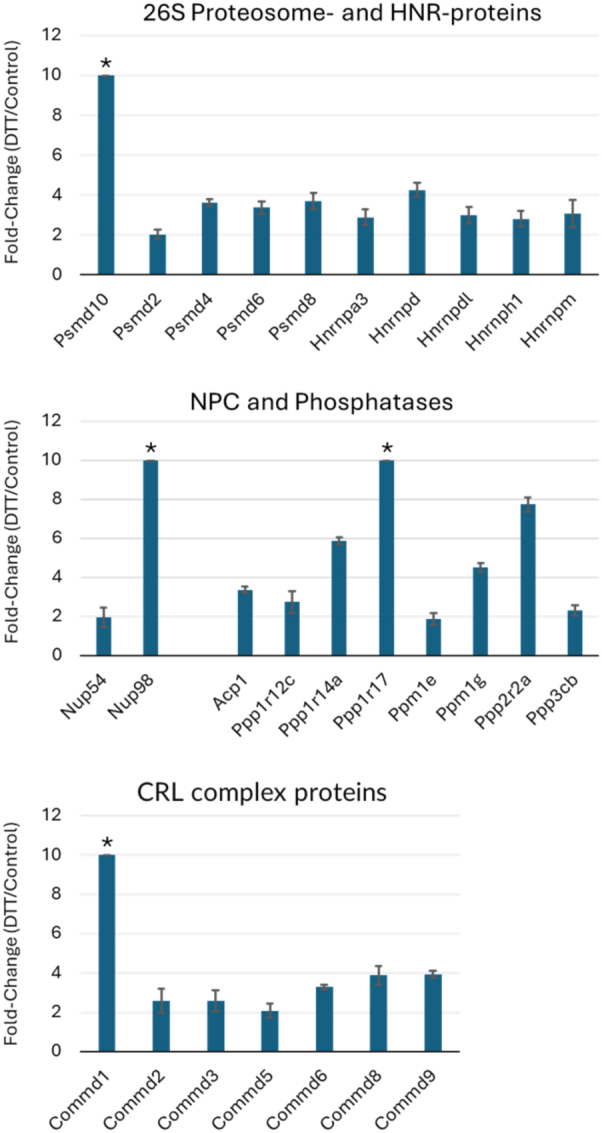


**FIGURE∼2:**
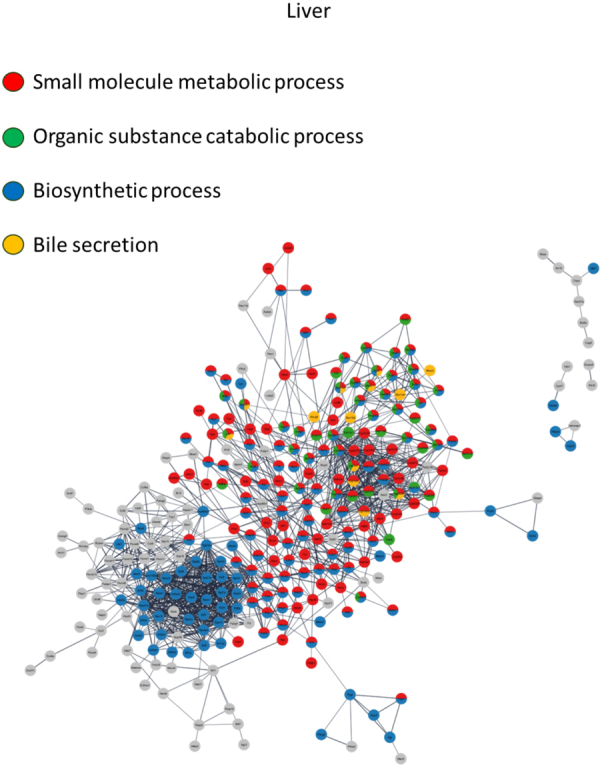


**FIGURE∼3:**
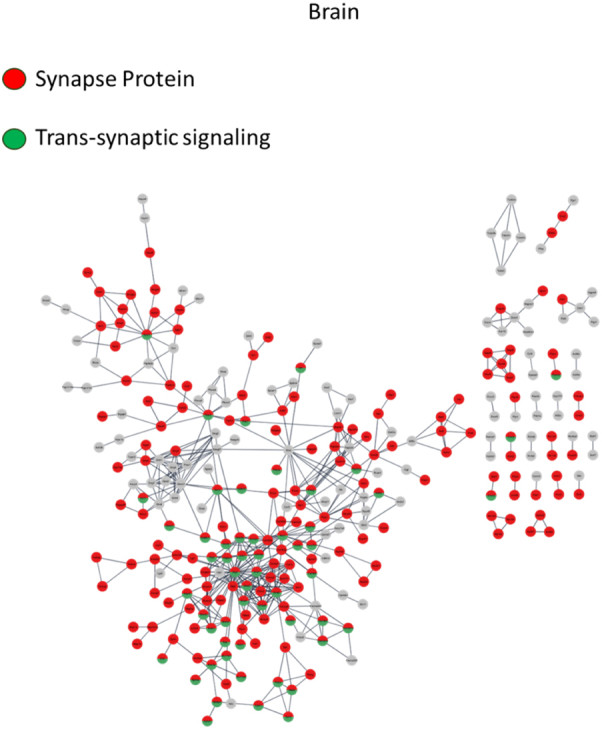


**FIGURE∼4:**
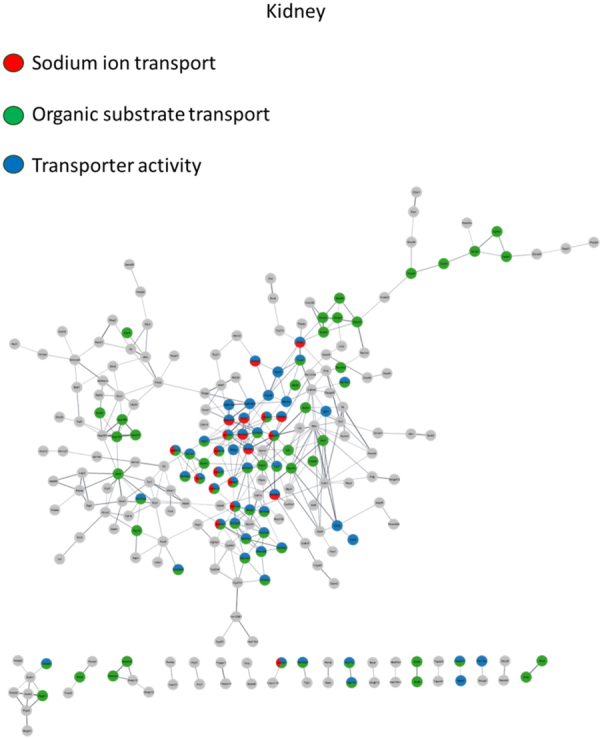


## Supplementary Data

DIABET∼1

Supplementary file

Mouse Brain Palm Validation

Mouse Brain Palm Proteome

Mouse Organ Tissue Palm Proteome

Acyl-RAC_SDC-ACE-Comparison
