# Supplementary material for: Enrichment of Cysteine S-palmitoylated Peptides Using Sodium Deoxycholate Acid Precipitation
Source: Mol Cell Proteomics. 2025 Oct 16;25(2):101218. doi: 10.1016/j.mcpro.2025.101218 (PMC12874130; doi:10.1016/j.mcpro.2025.101218)
Supplement: Mouse Organ Tissue Palm Proteome [file mmc5.pdf]

zDHHC1

Proteome discoverer coverage

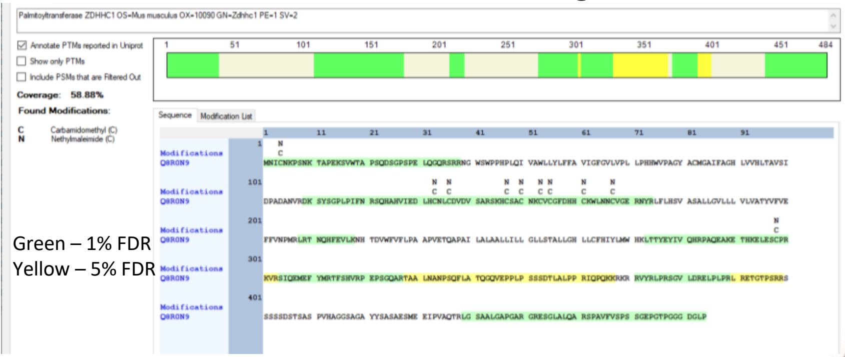

Green – 1% FDR
Yellow – 5% FDR

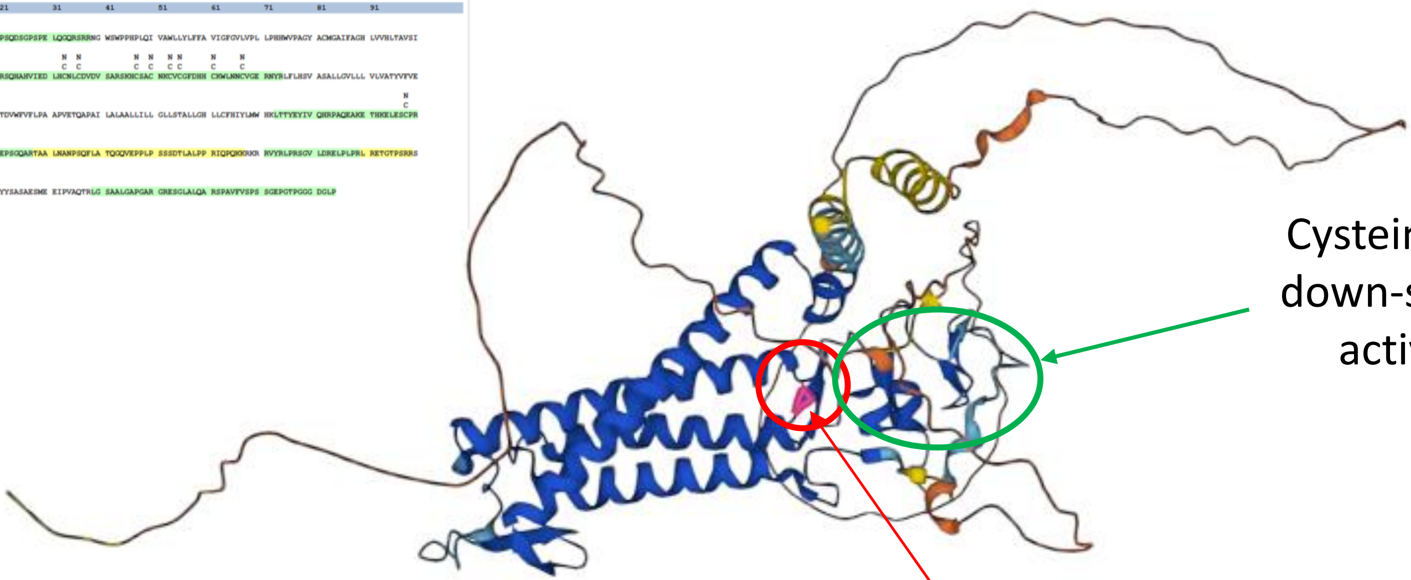

Cysteine stack - down-stream to active site

Helical - transmembrane

C_{161} – active site

HCNLCDVDVSARSKHCSACNKCVCGFDHHC_{161}KWLNNCVG

= zDHHC motif

Underscore = Identified palmitoylation sites

Proteome discoverer coverage

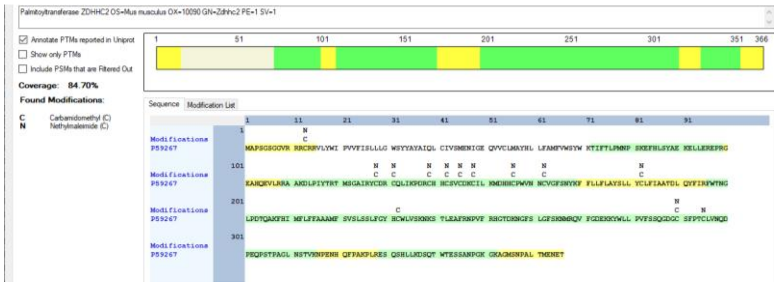

zDHHC2

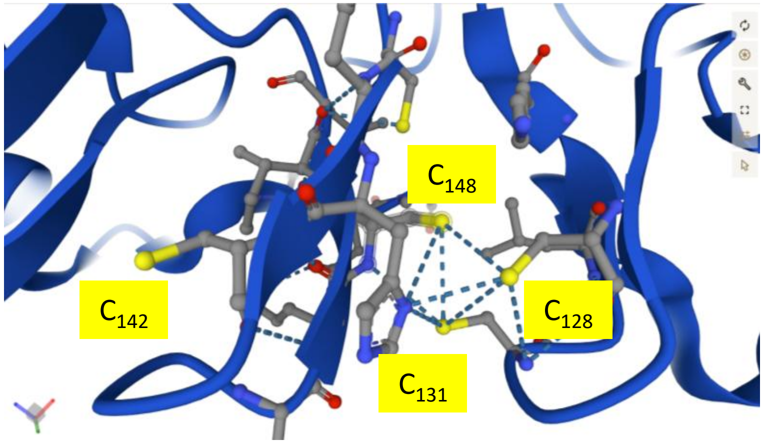

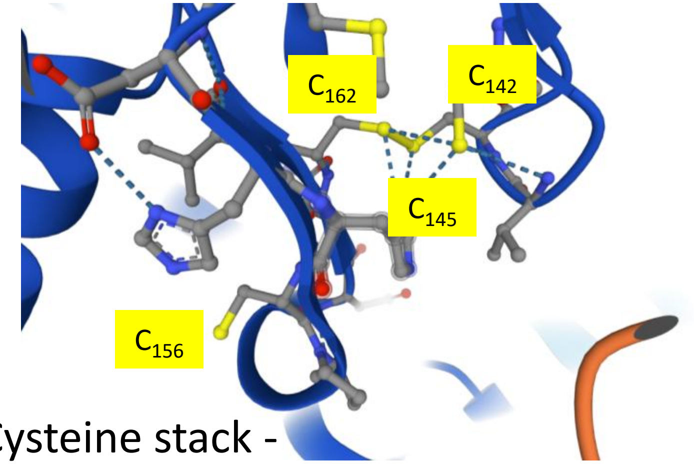

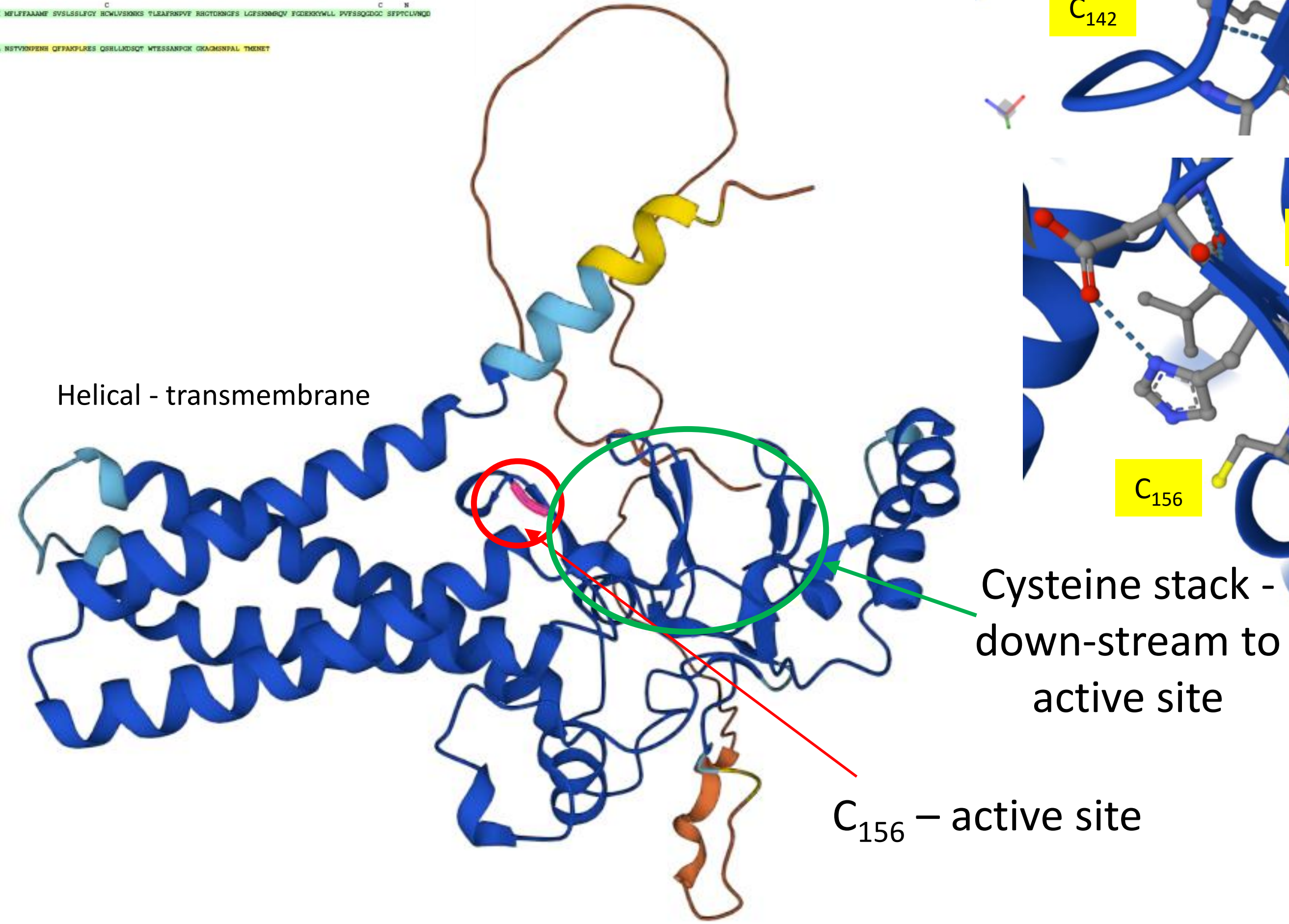

YCDRCQLIKPDRCHHCSVCDKCILKMDHHC156PWVNNCVG

Proteome discoverer coverage

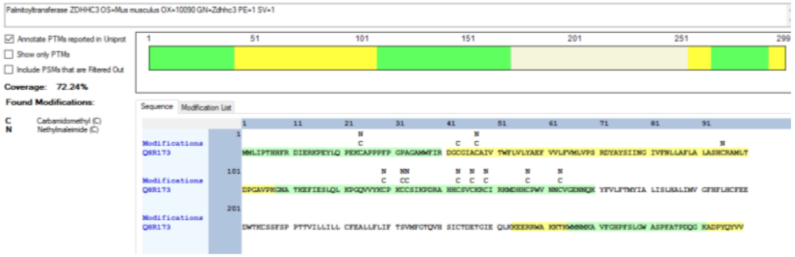

zDHHC3

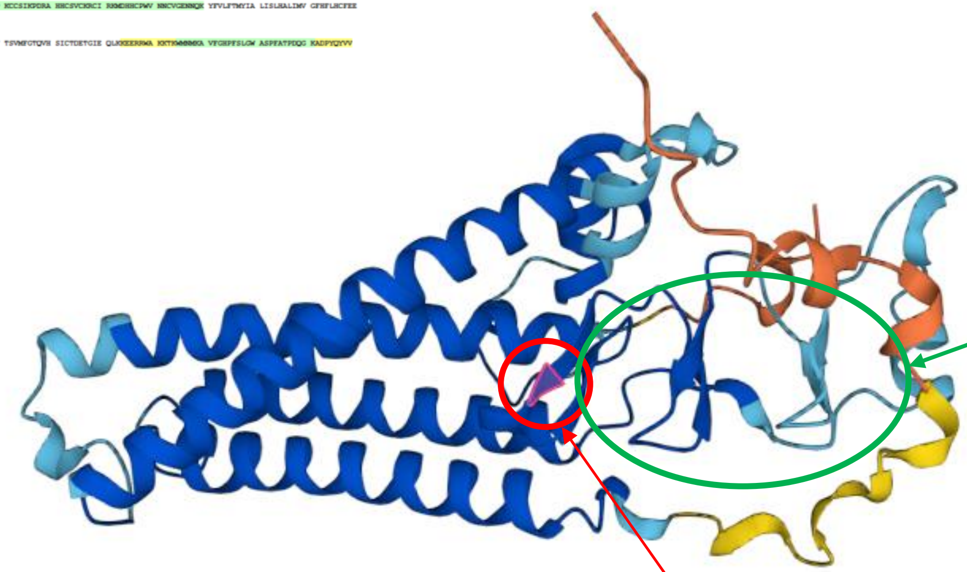

Cysteine stack - down-stream to active site

C_{157} – active site

K<u>C</u>PK<u>CC</u>SIKPDRAHH<u>C</u>SV<u>C</u>KR<u>C</u>IRKM<u>DHHC</u>$_{157}$PWVNN<u>C</u>VG

zDHHC4

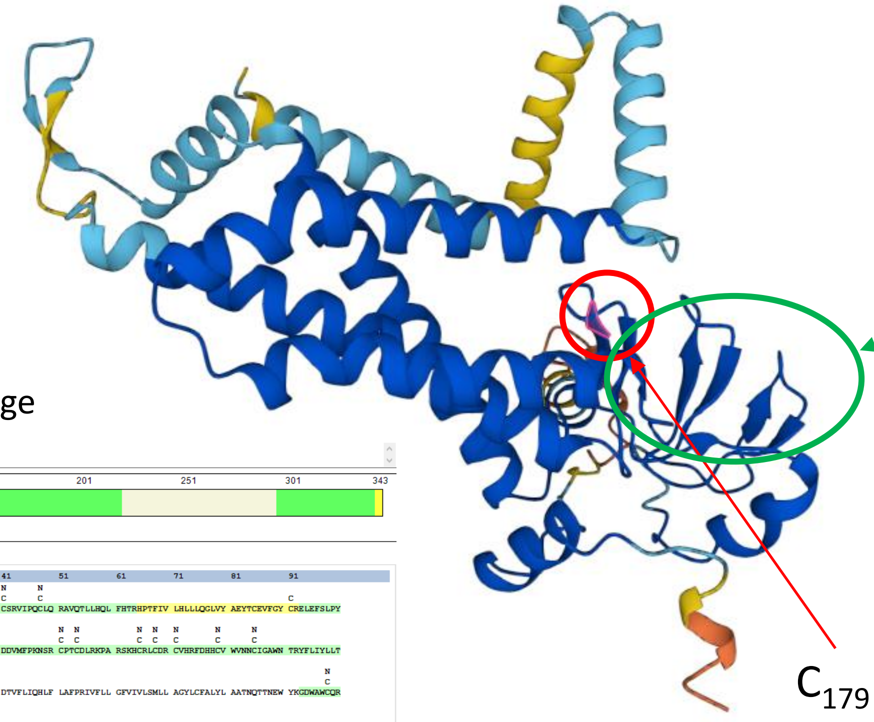

Cysteine stack - down-stream to active site

C_{179} – active site

Proteome discoverer coverage

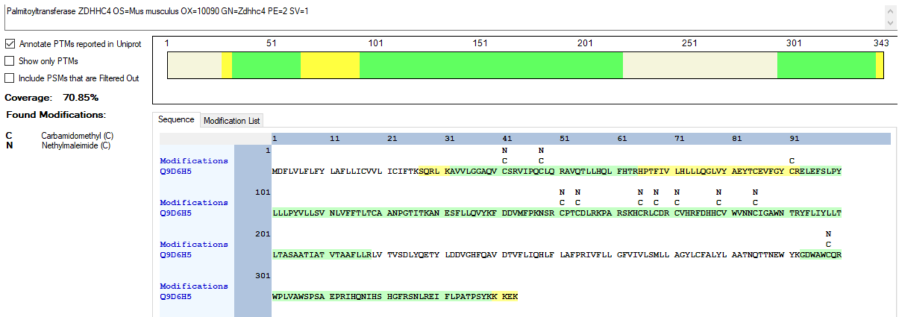

R<u>C</u>PT<u>C</u>DLRKPARSKH<u>C</u>RL<u>C</u>DR<u>C</u>VHRF<u>DHHC</u>$_{179}$VWVNN<u>C</u>IG

zDHHC5

$C_{236}C_{237}$-7x-C_{245}

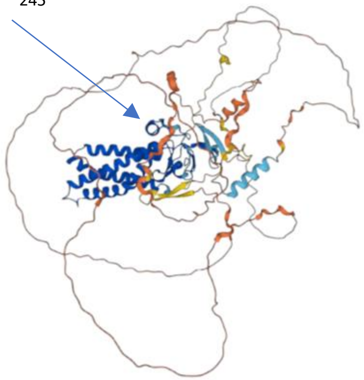

Proteome discoverer coverage

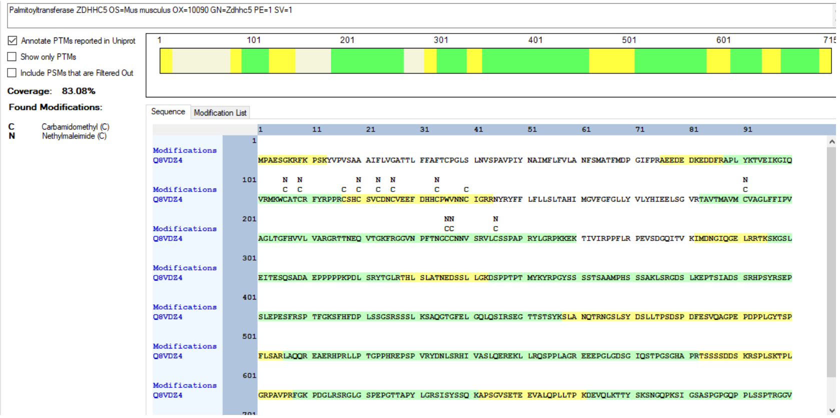

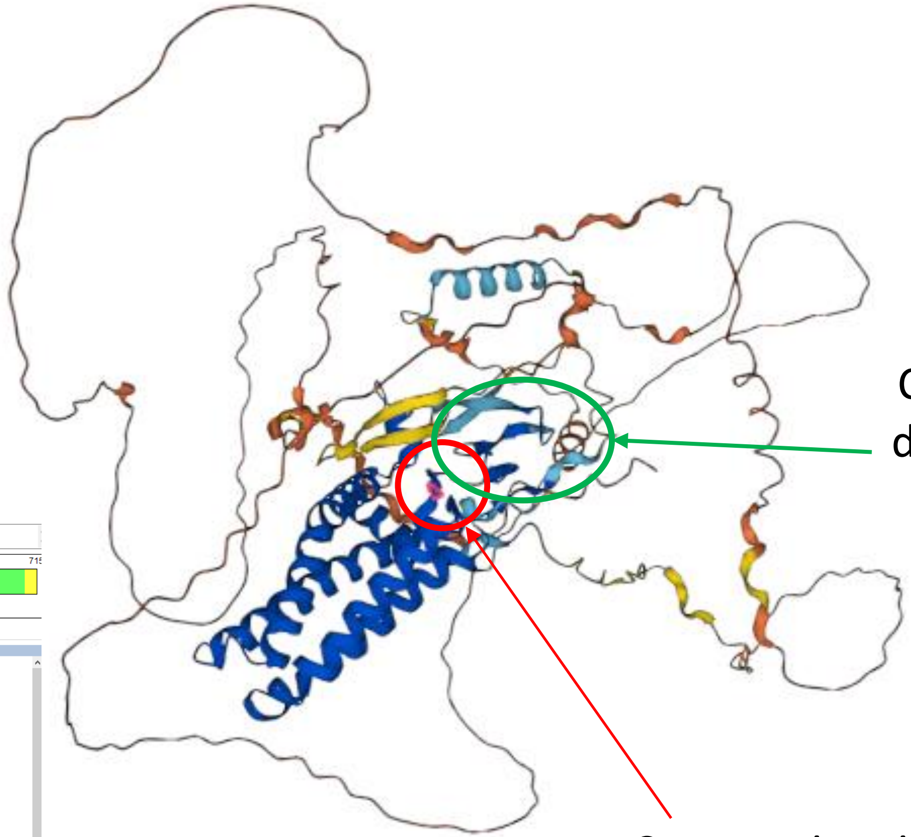

Cysteine stack - down-stream to active site

C_{134} – active site

WCATCRFYRPPRCSHCSVCDNCVEEFDHHC_{134}PWVNNCIG

zDHHC6

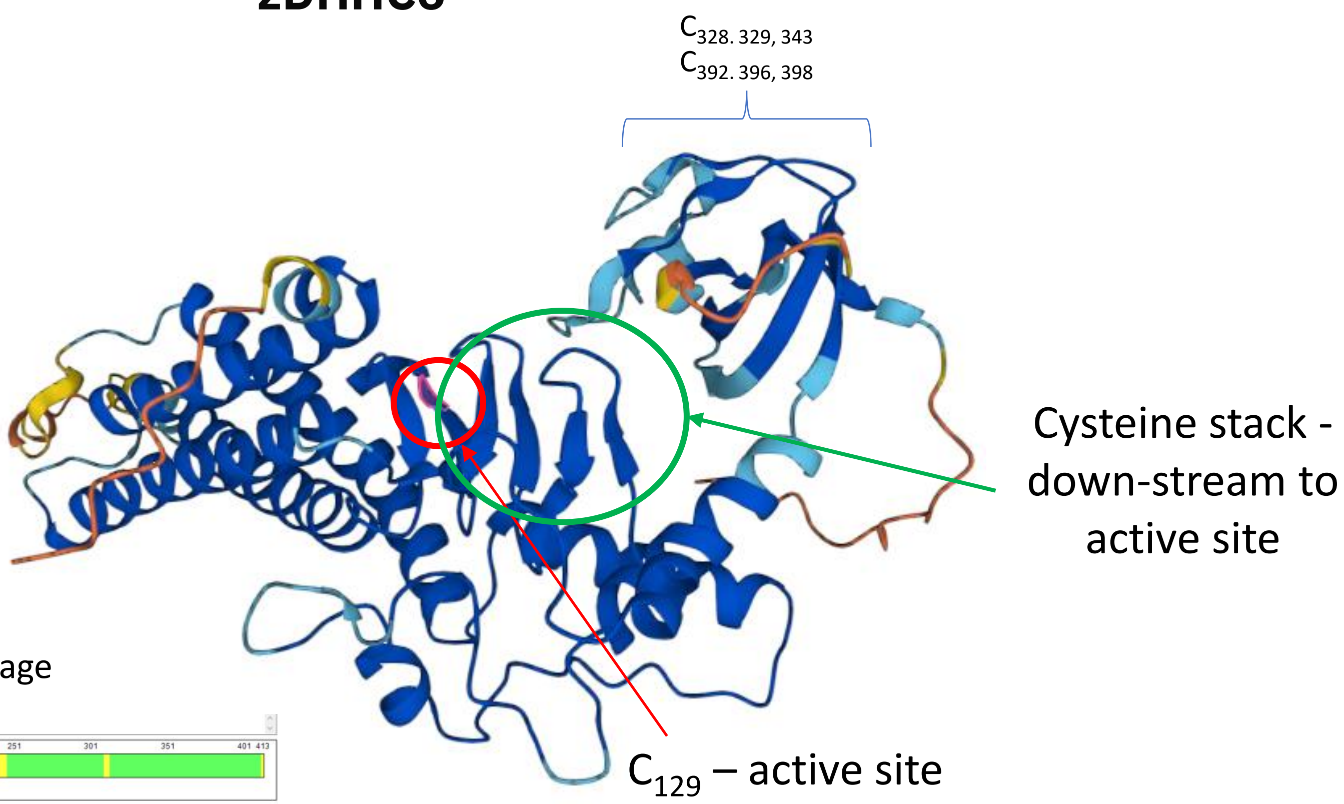

Proteome discoverer coverage

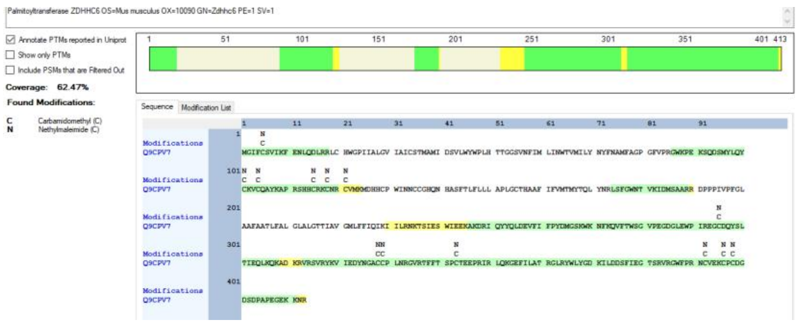

YCKVCQAYKAPRSHHCRKCNRCVMKMDHHC$_{129}$PWINNCCG

zDHHC7

Proteome discoverer coverage

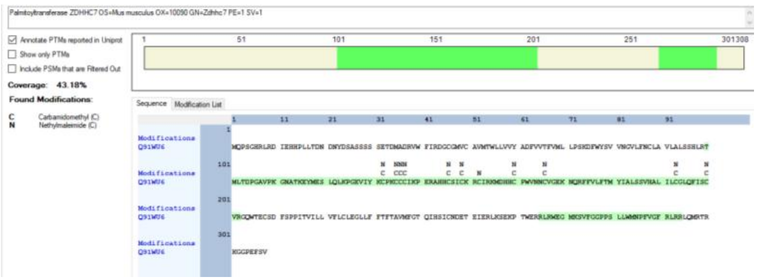

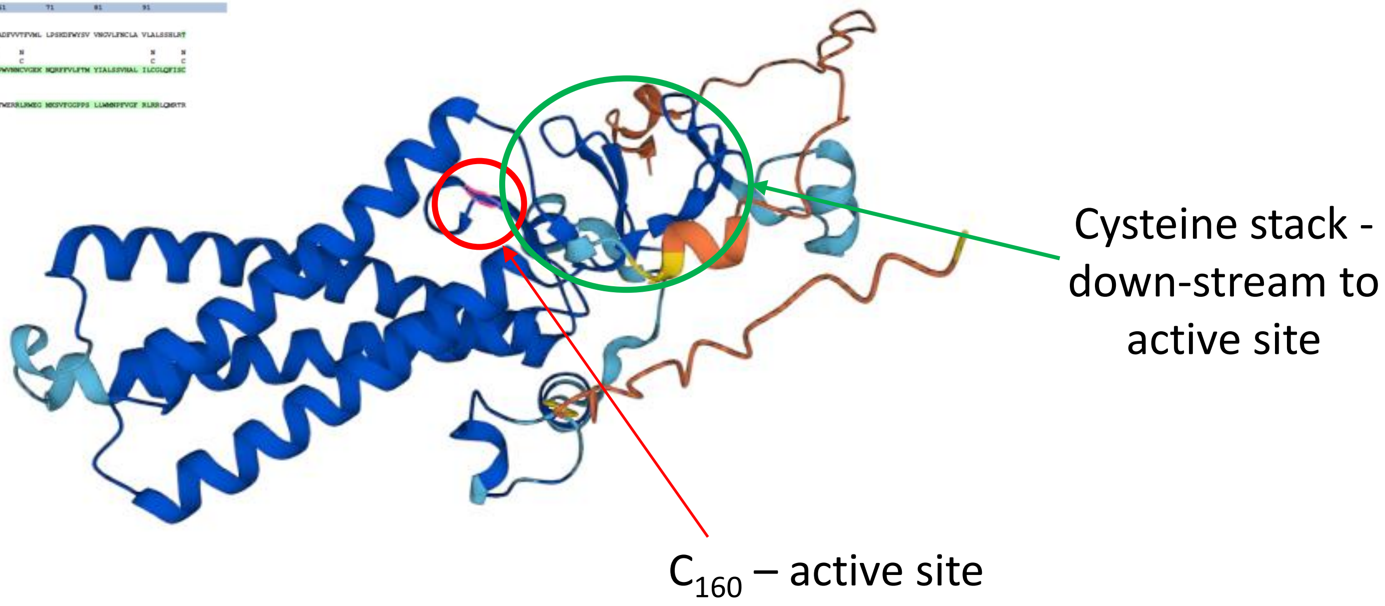

KCPKCCCIKPERAHHCSICKRCIRKMDHHC$_{160}$PWVNNCVG

zDHHC8

Proteome discoverer coverage

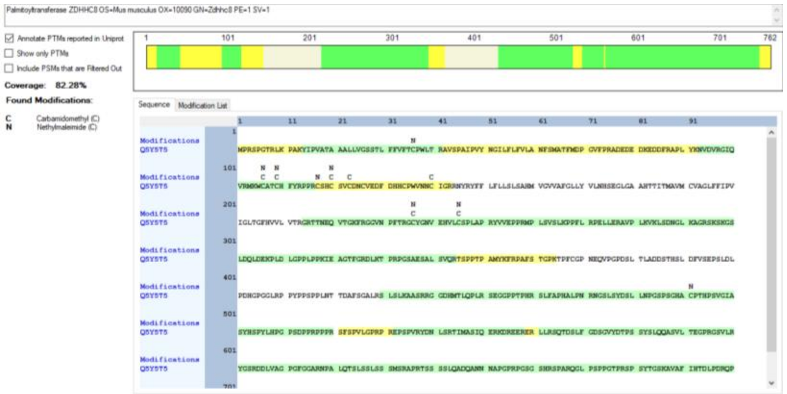

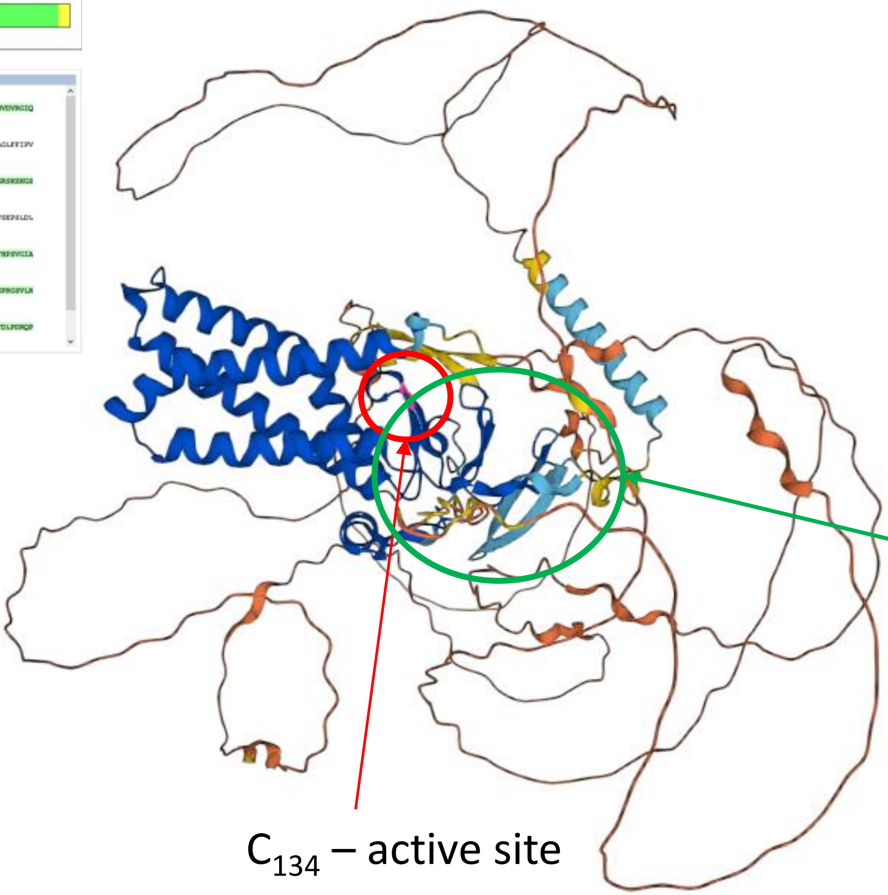

WCATCHFYRPPRCSHCSVCDNCVEDFDHHC$_{134}$PWVNNCIG

zDHHC9

Proteome discoverer coverage

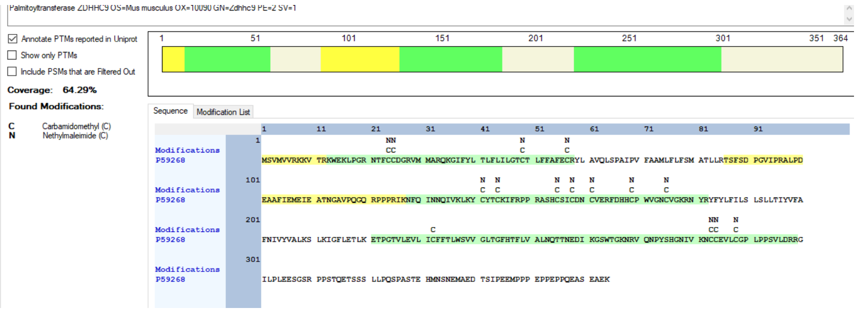

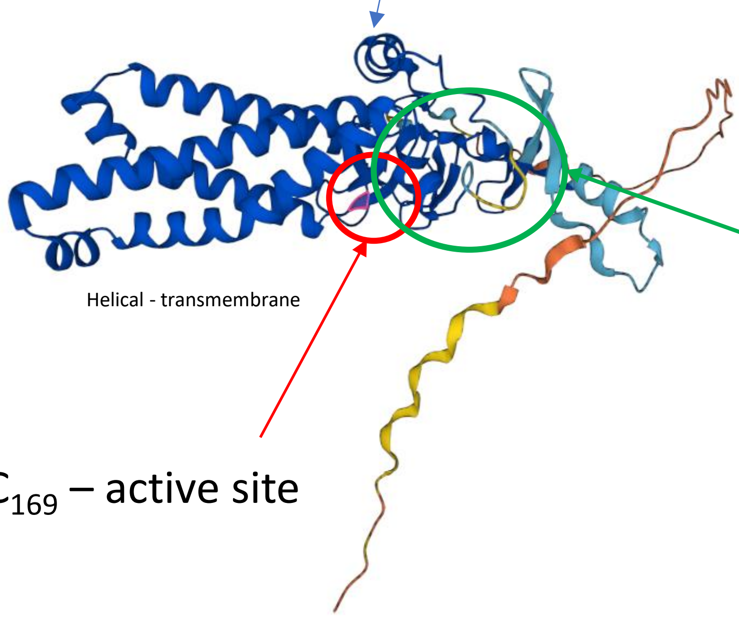

YCYTCKIFRPPRASHCSICDNCVERFDHHC_{169}PWVGNCVG

zDHHC11

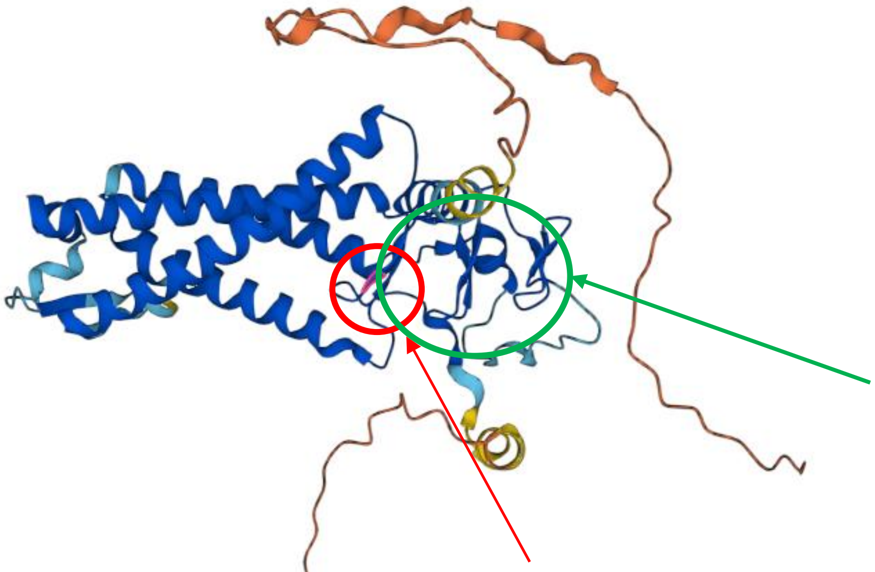

Proteome discoverer coverage

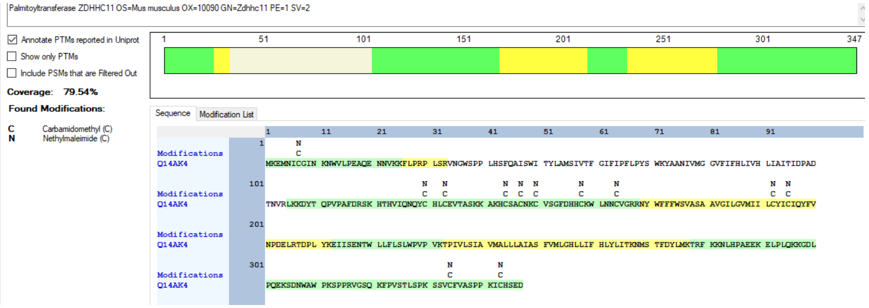

YCHLCEVTASKKAKHCSACNKCVSGFDHHC$_{158}$KWLNNCVG

zDHHC12

Proteome discoverer coverage

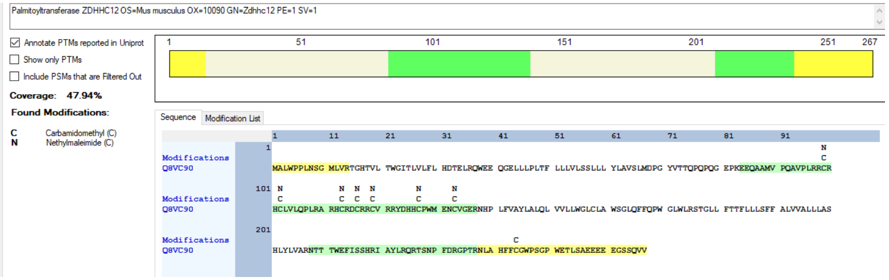

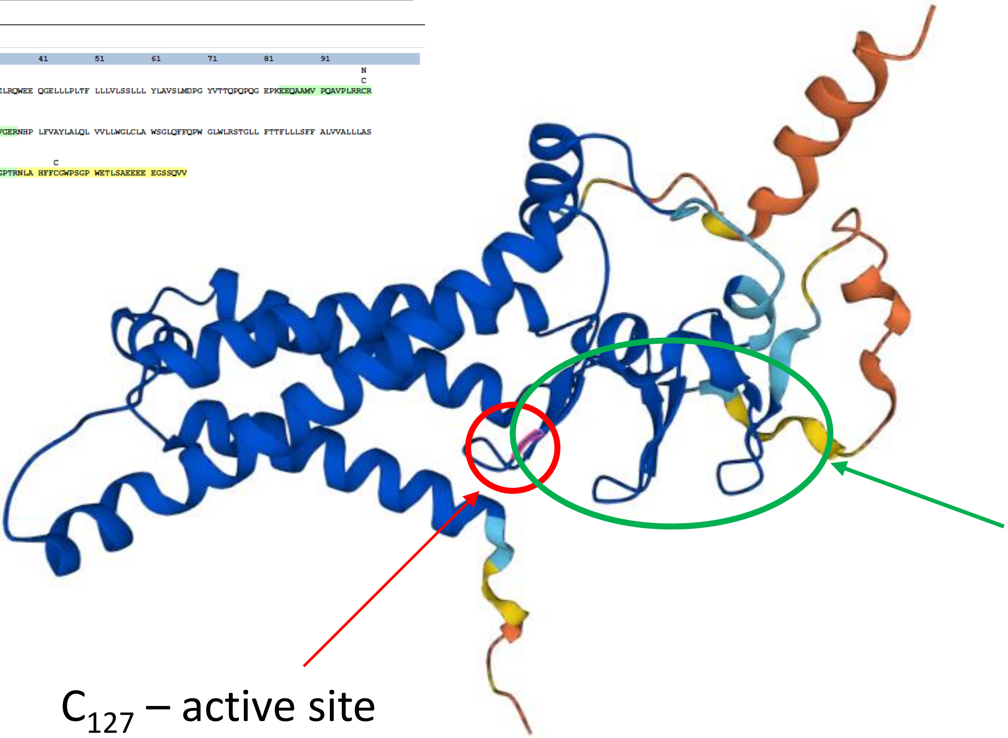

Cysteine stack - down-stream to active site

C_{127} – active site

RCRHCLVLQPLRARHCRDCRRCVRRYDHHC_{127}PWMENCVG

zDHHC13

Proteome discoverer coverage

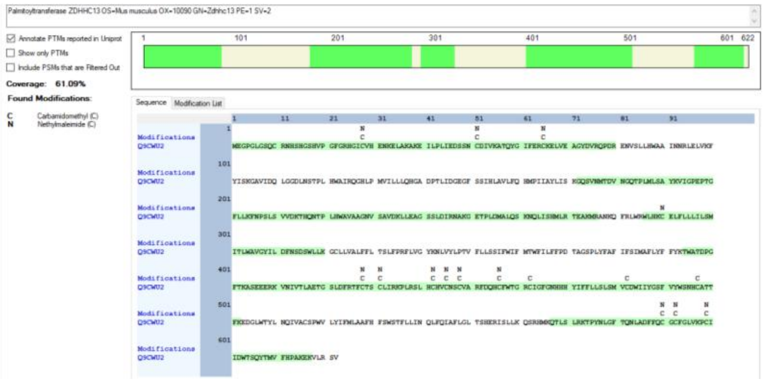

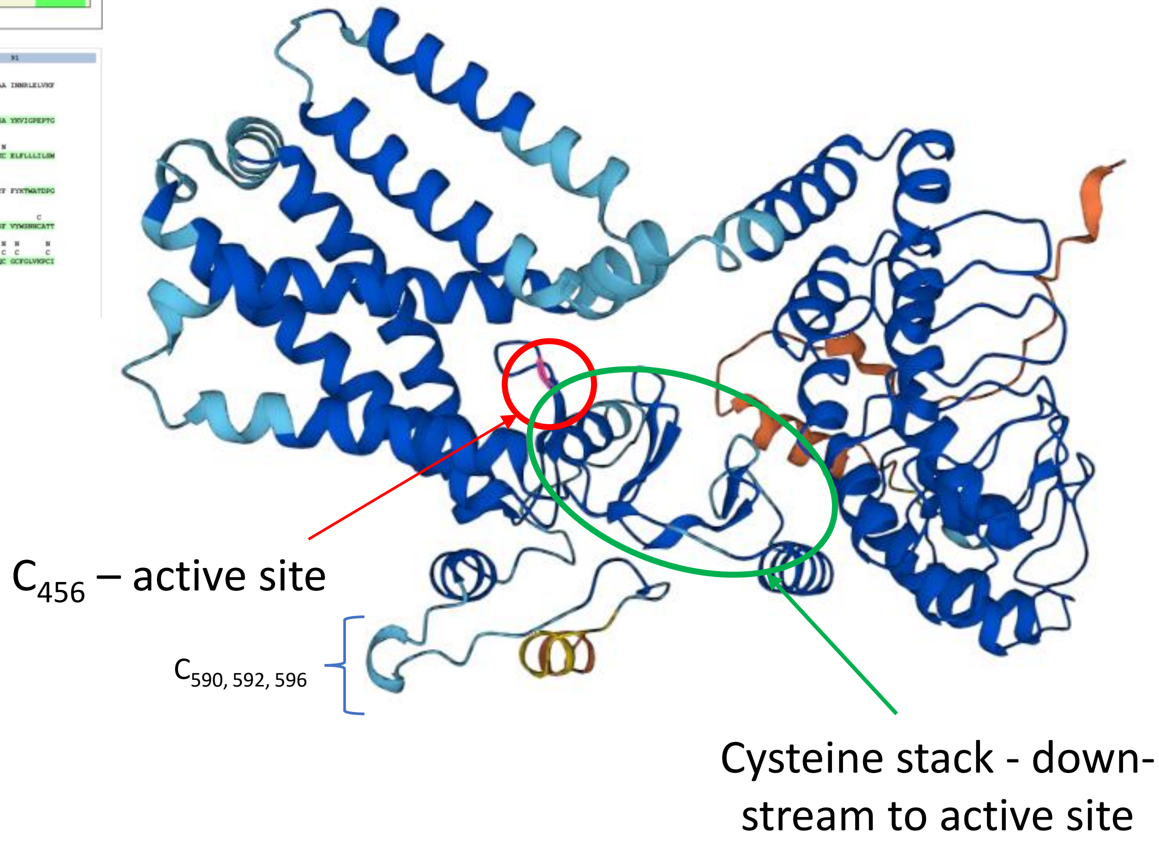

FCTSCLIRKPLRSLHCHVCNSCVARFDQHC$_{456}$FWTGRCIG

zDHHC14

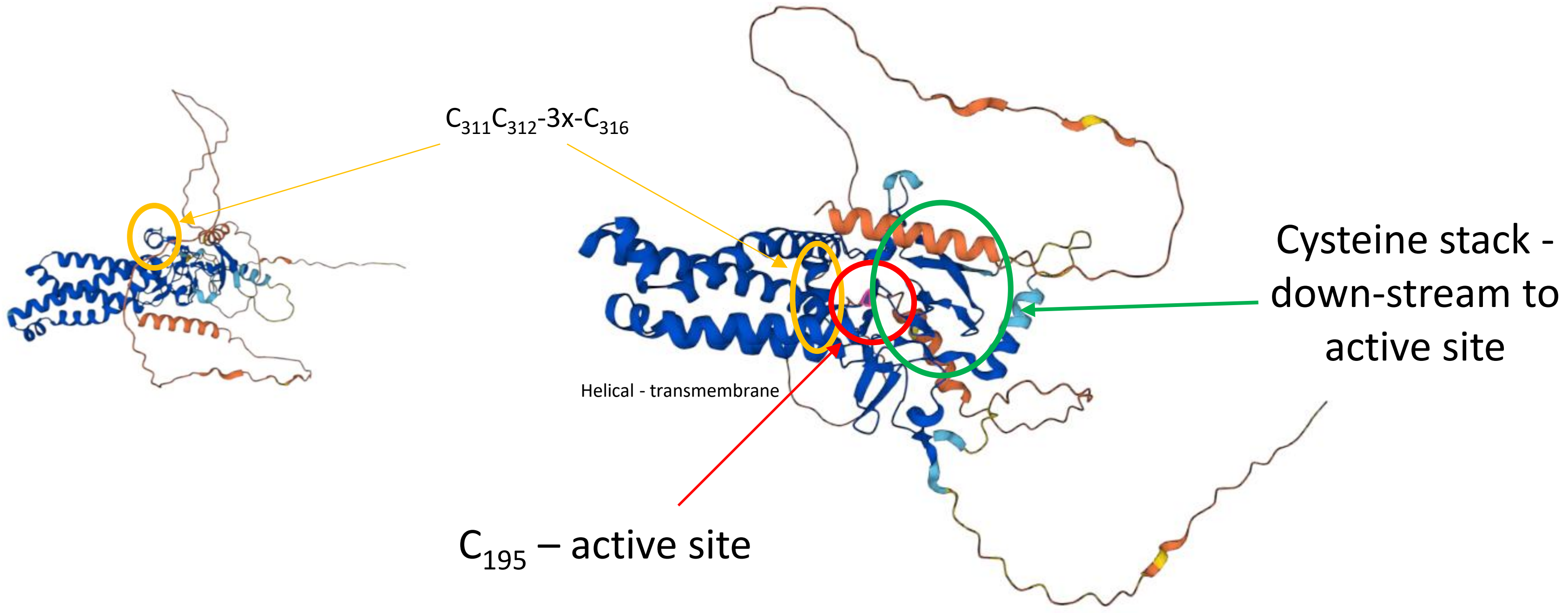

Proteome discoverer coverage

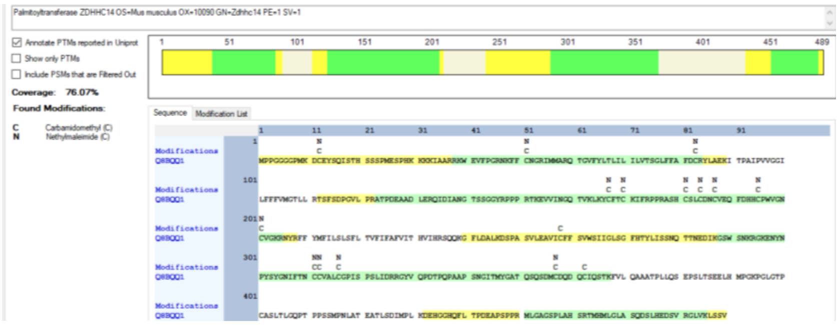

YCFTCKIFRPPRASHCSLCDNCVEQFDHHC$_{195}$PWVGNCVG

zDHHC15

Proteome discoverer coverage

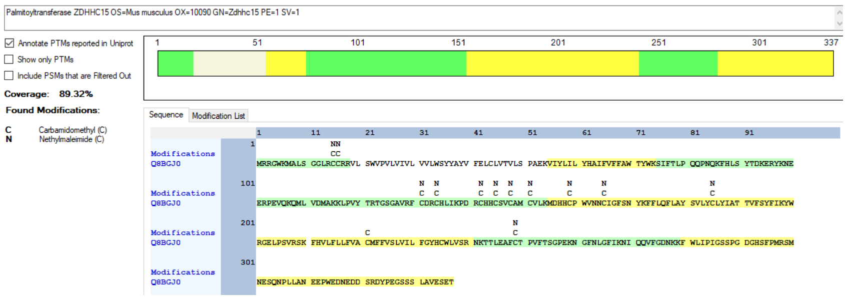

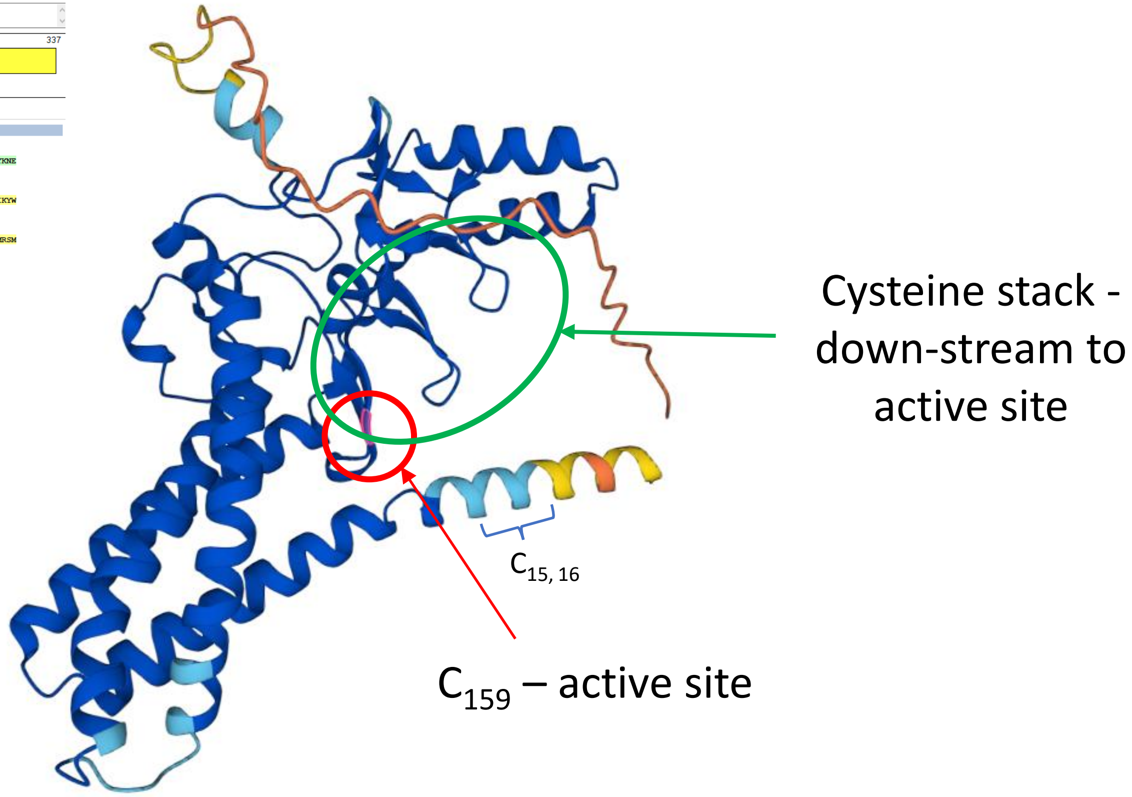

FCDRCHLIKPDRCHHCSVCAMCVLKMDHHC$_{159}$PWVNNCIG

zDHHC16

Proteome discoverer coverage

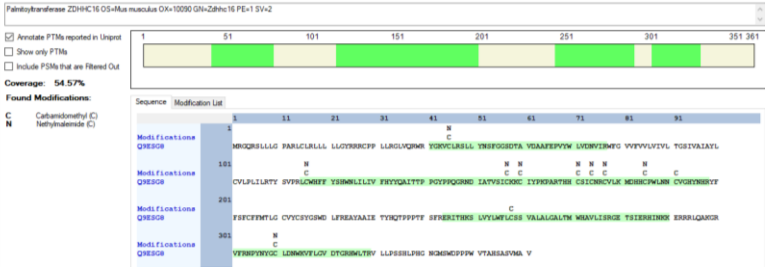

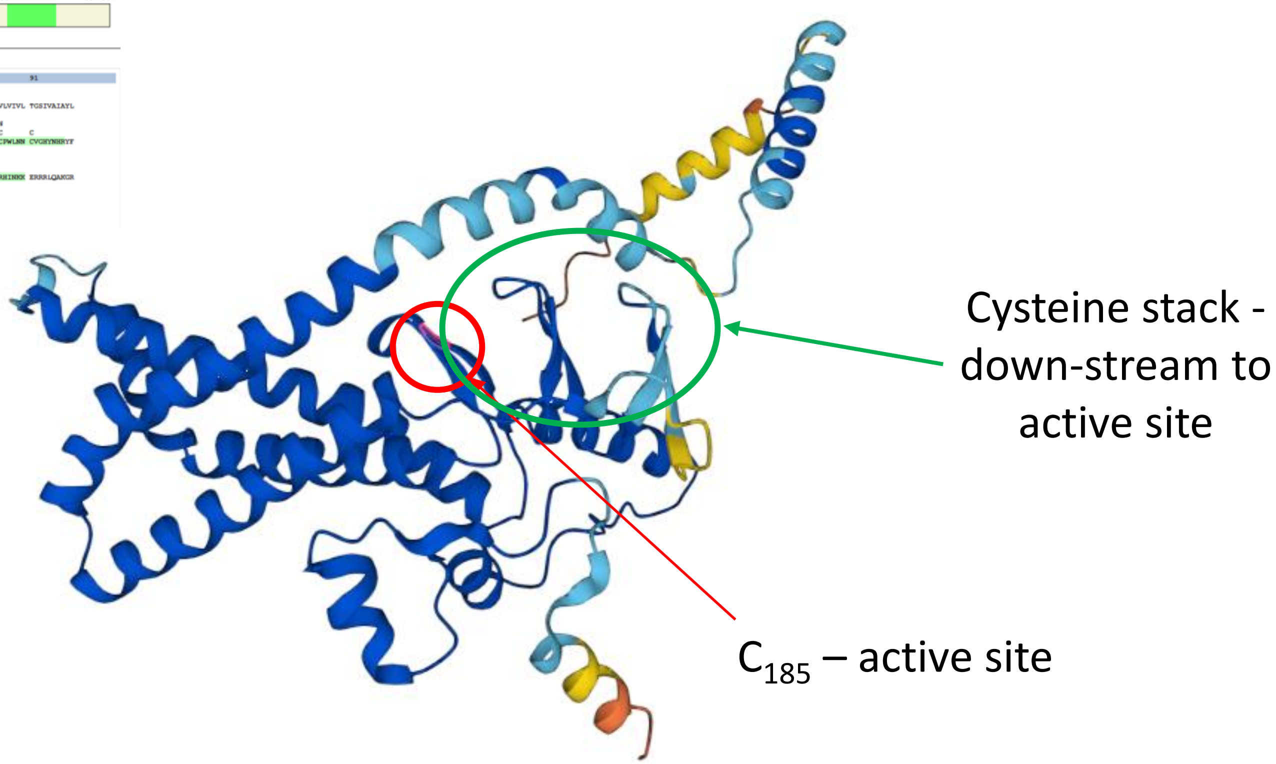

ICKKCIYPKPARTHHCSICNRCVLKMDHHC_{185}PWLNNCVG

zDHHC17

Proteome discoverer coverage

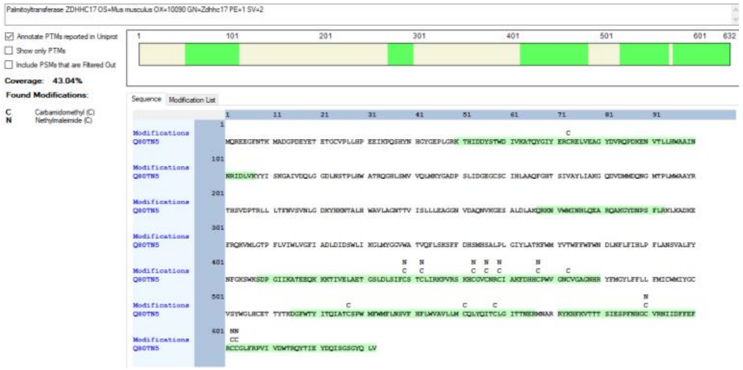

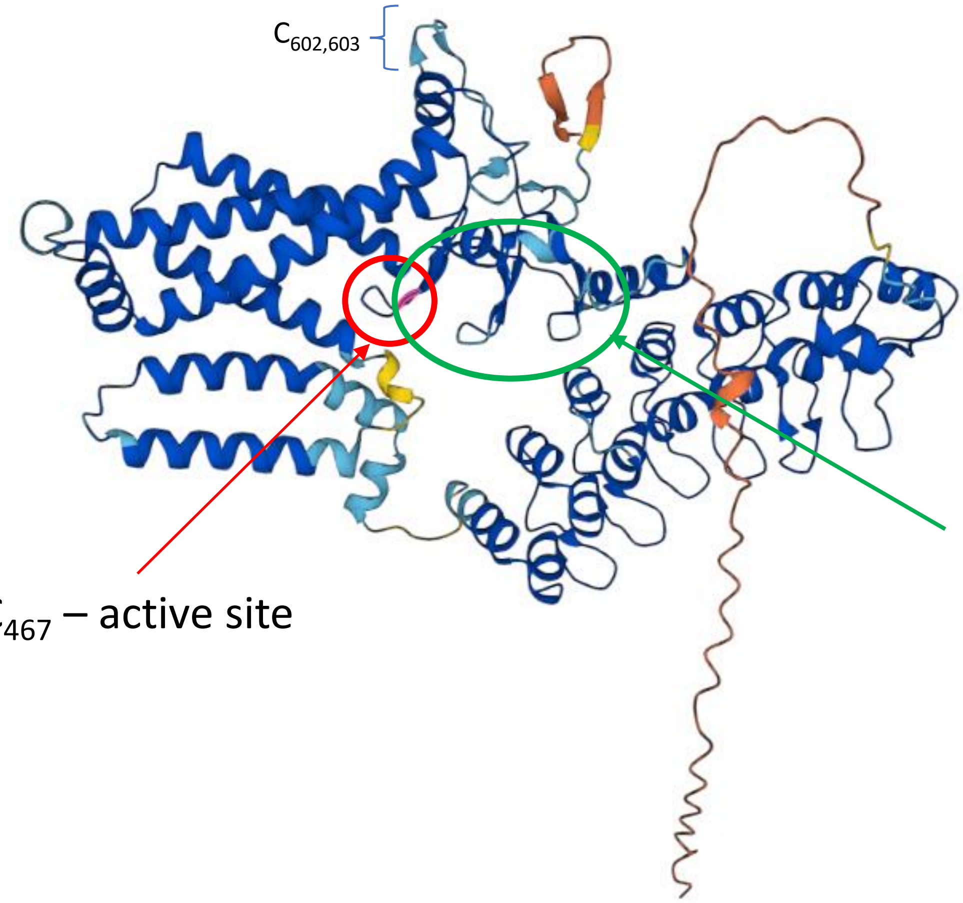

FCSTCLIRKPVRSKHCGVCNRCIAKFDHHC$_{467}$PWVGNCVG

zDHHC18

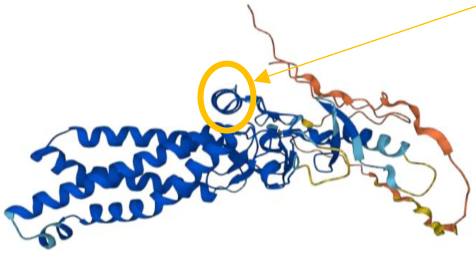

$C_{331}C_{332}$-4x-C_{336}

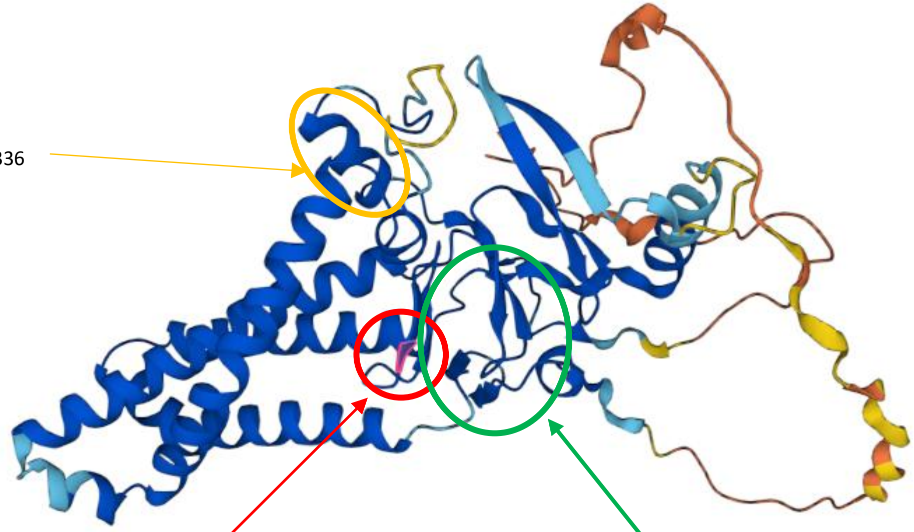

C_{214} – active site

Cysteine stack - down-stream to active site

Proteome discoverer coverage

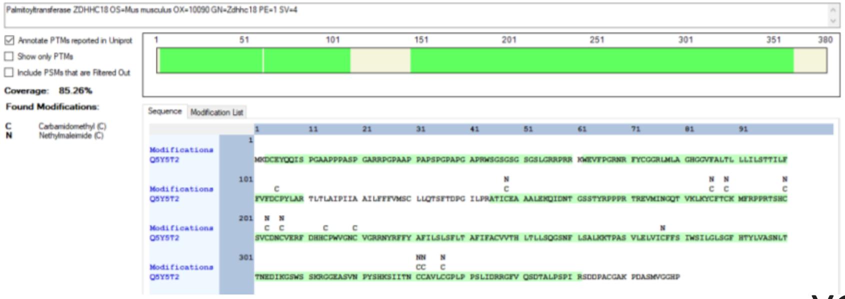

Y<u>C</u>FT<u>C</u>KMFRPPRTSH<u>C</u>SV<u>C</u>DN<u>C</u>VERF<u>DHHC_{214}</u>PWVGN<u>C</u>VG

zDHHC19

Proteome discoverer coverage

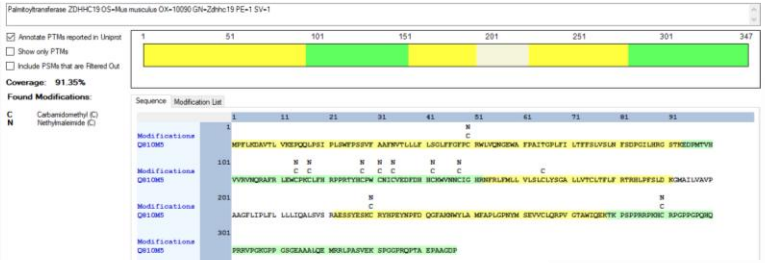

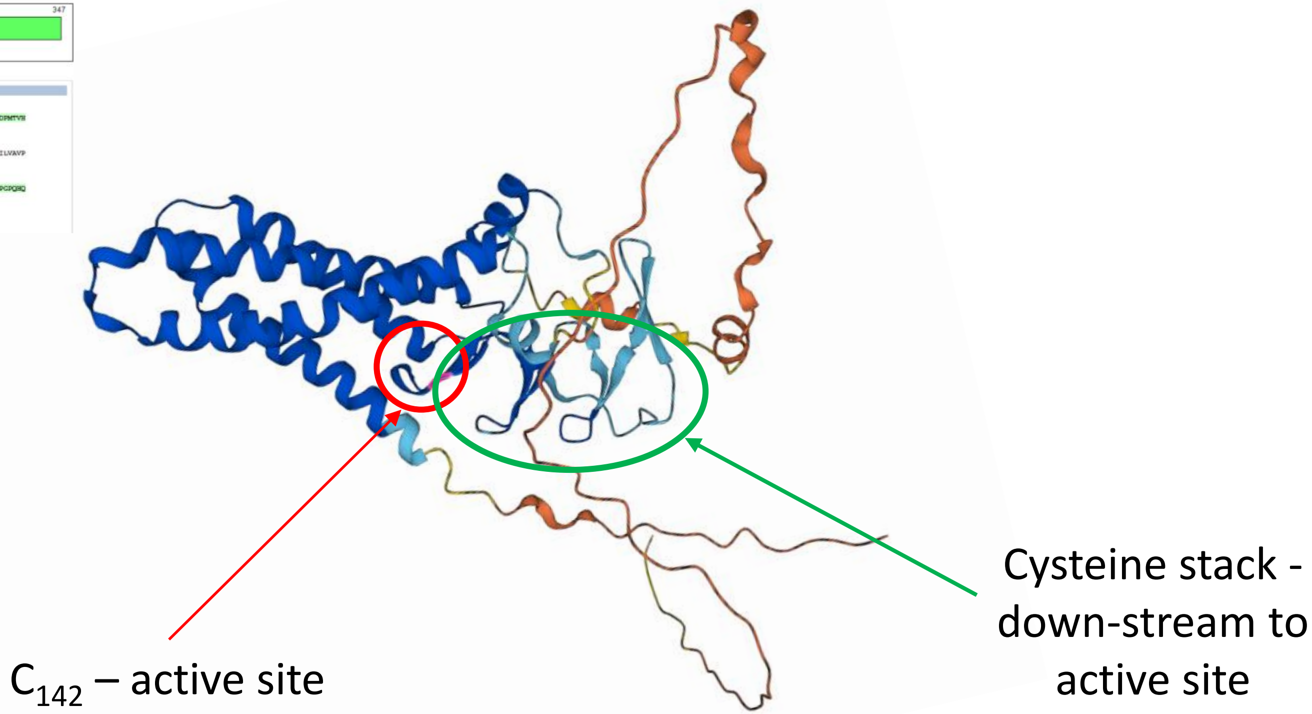

WCPKCLFHRPPRTYHCPWCNICVEDFDHHC$_{142}$KWVNNCIG

zDHHC20

Proteome discoverer coverage

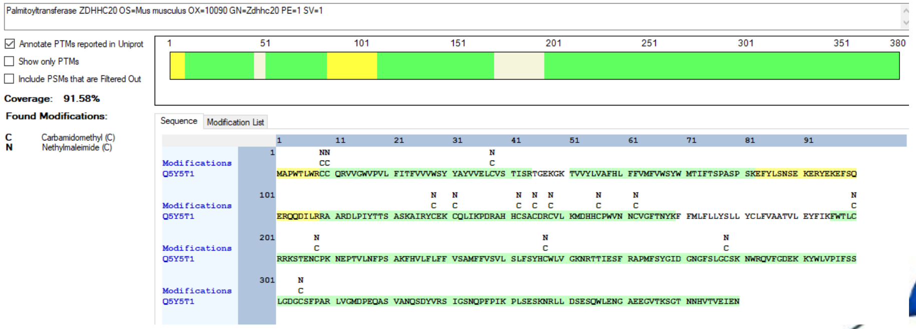

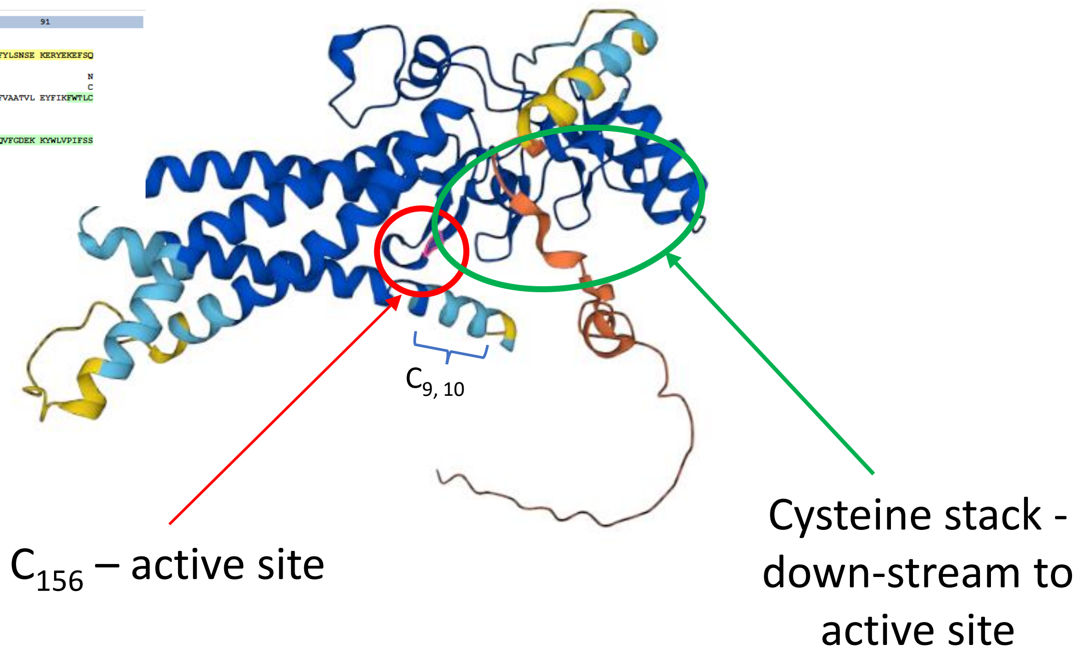

YCEKCQLIKPDRAHHCSACDRCVLKMDHHC$_{156}$PWVNNCVG

zDHHC21

Proteome discoverer coverage

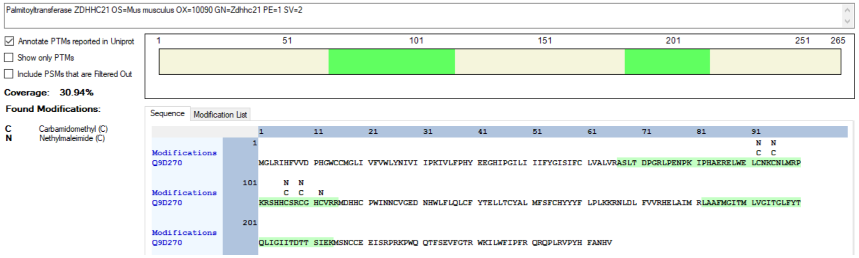

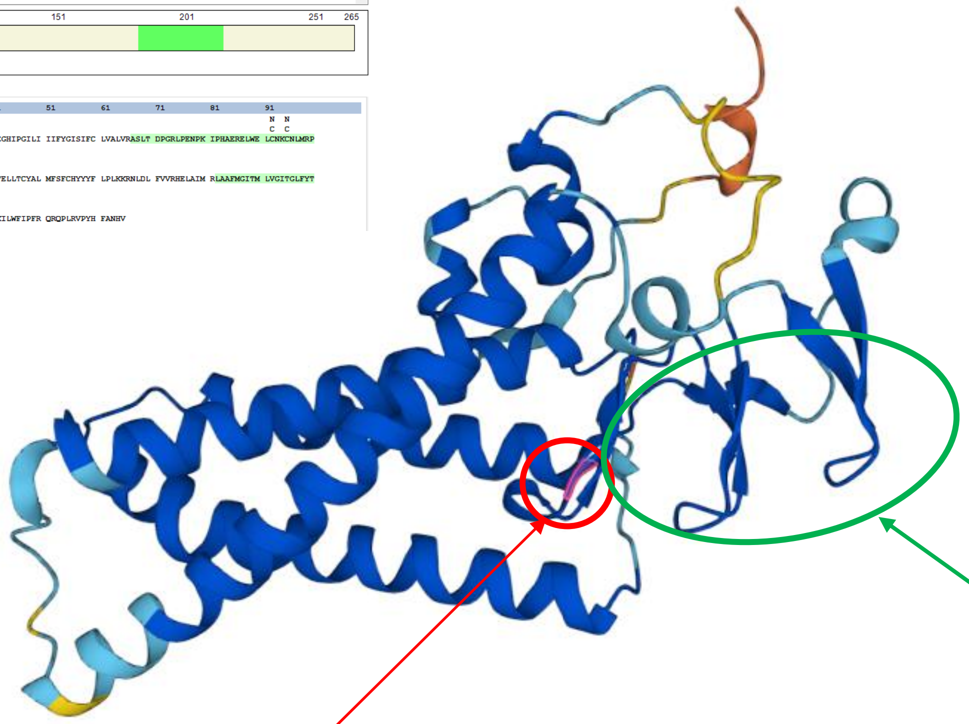

C_{120} – active site

Cysteine stack - down-stream to active site

LCNKCNLMRPKRSHHCSRCGHCVRRMDHHC_{120}PWINNCVG

Proteome discoverer coverage

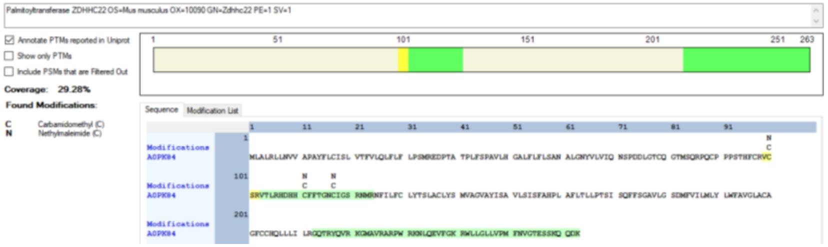

zDHHC22

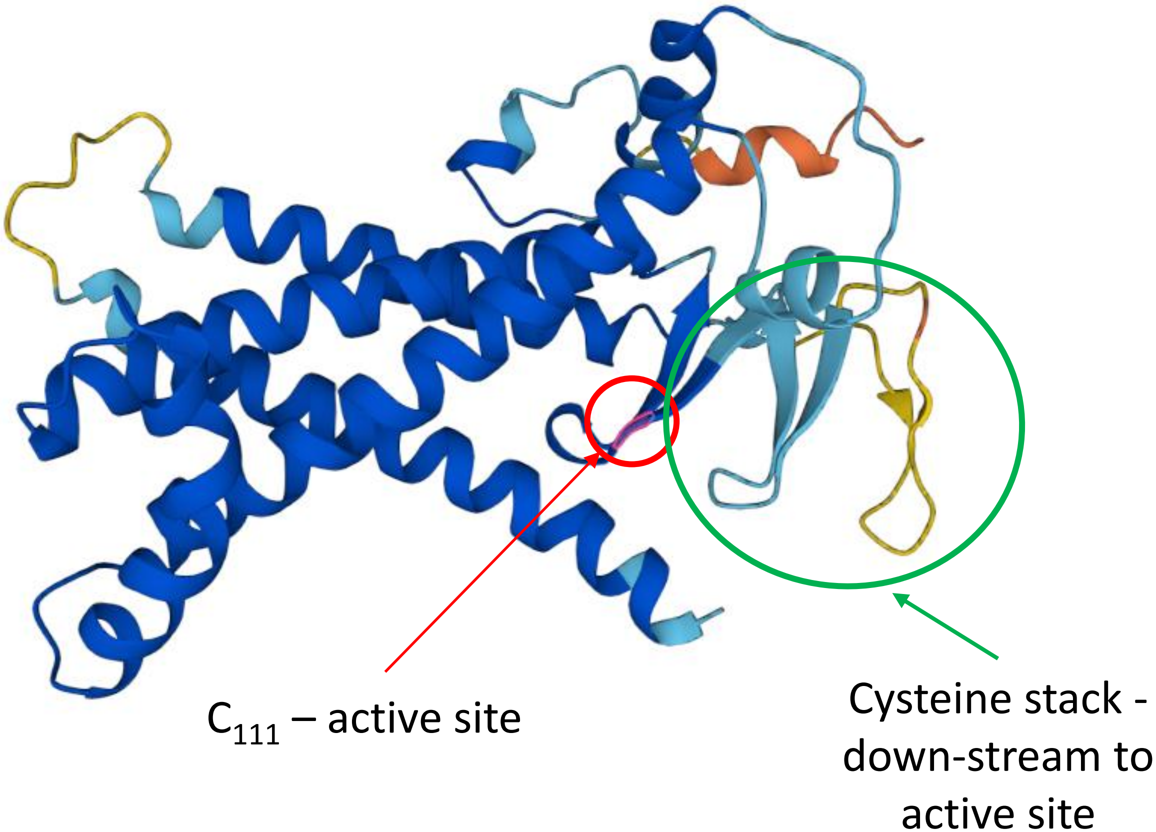

TMSQRPQCPPPSTHFCRVCSRVTLRHDHHC$_{111}$FFTGNCIG

Lack zDHHC consensus sequence

zDHHC23

Proteome discoverer coverage

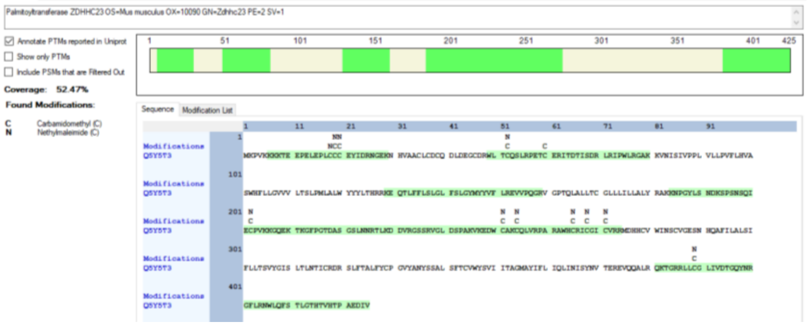

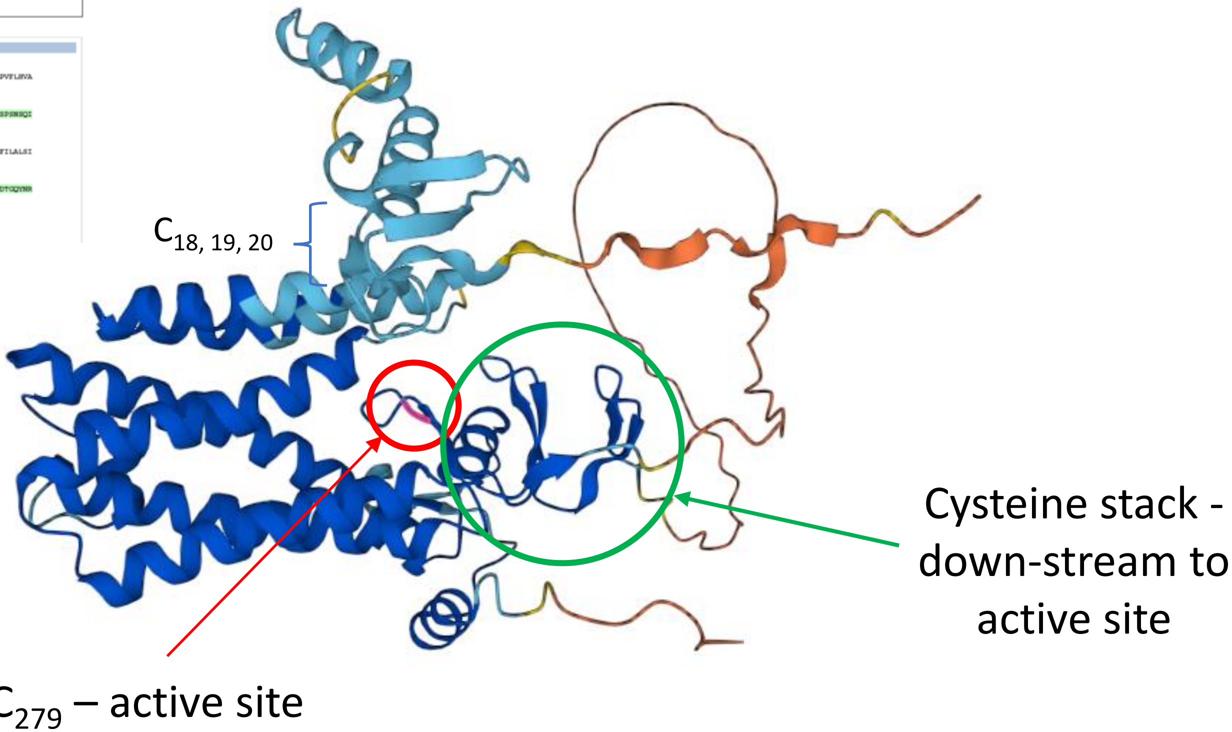

WC̲AKC̲QLVRPARAWHC̲RIC̲GIC̲VRRMD̲H̲H̲C̲$_{279}$VWINSC̲VG

zDHHC24

Proteome discoverer coverage

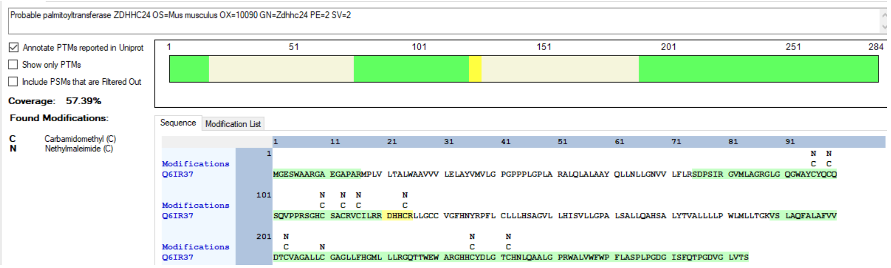

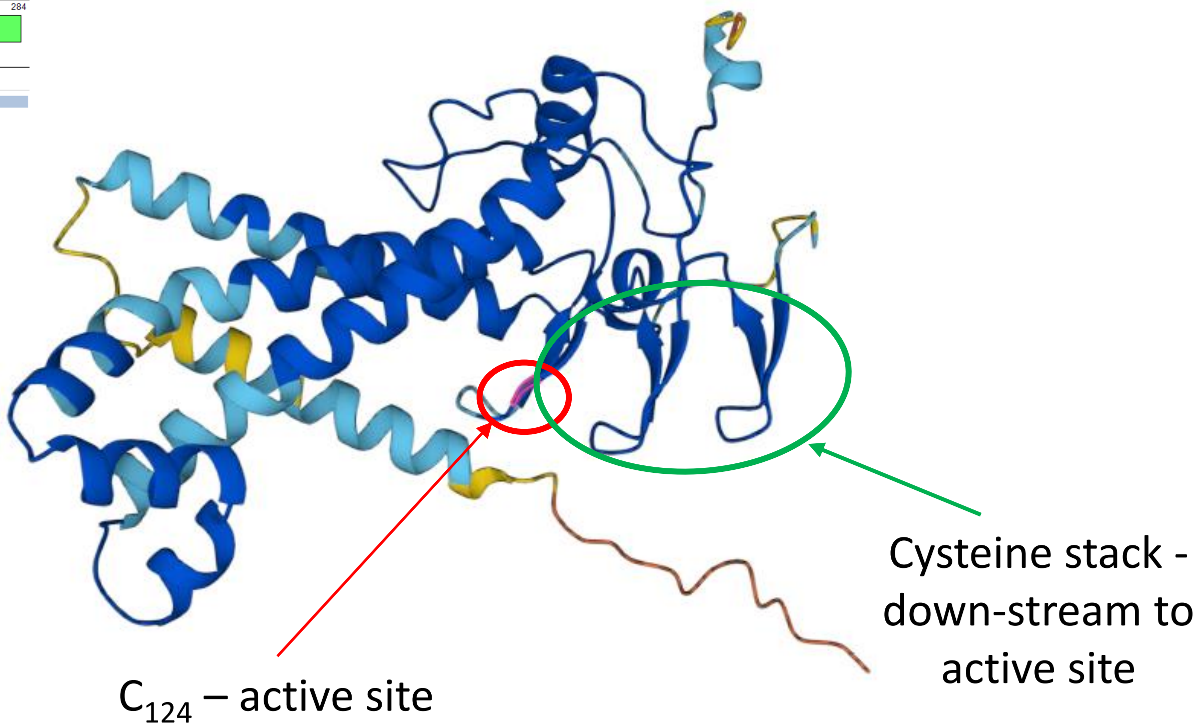

YCYQCQSQVPPRSGHCSACRVCILRRDHHC$_{124}$RLLGCCVG

zDHHC25

Proteome discoverer coverage

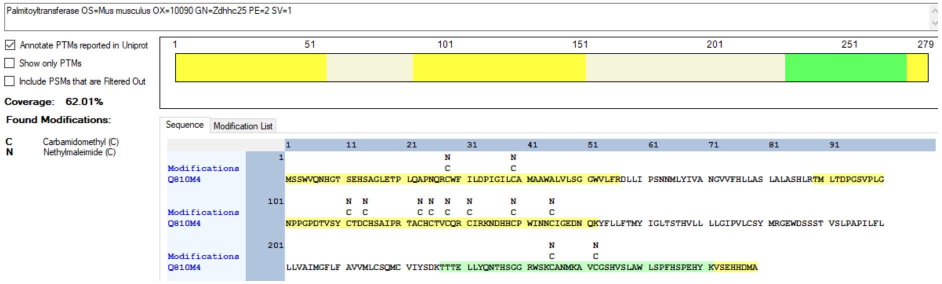

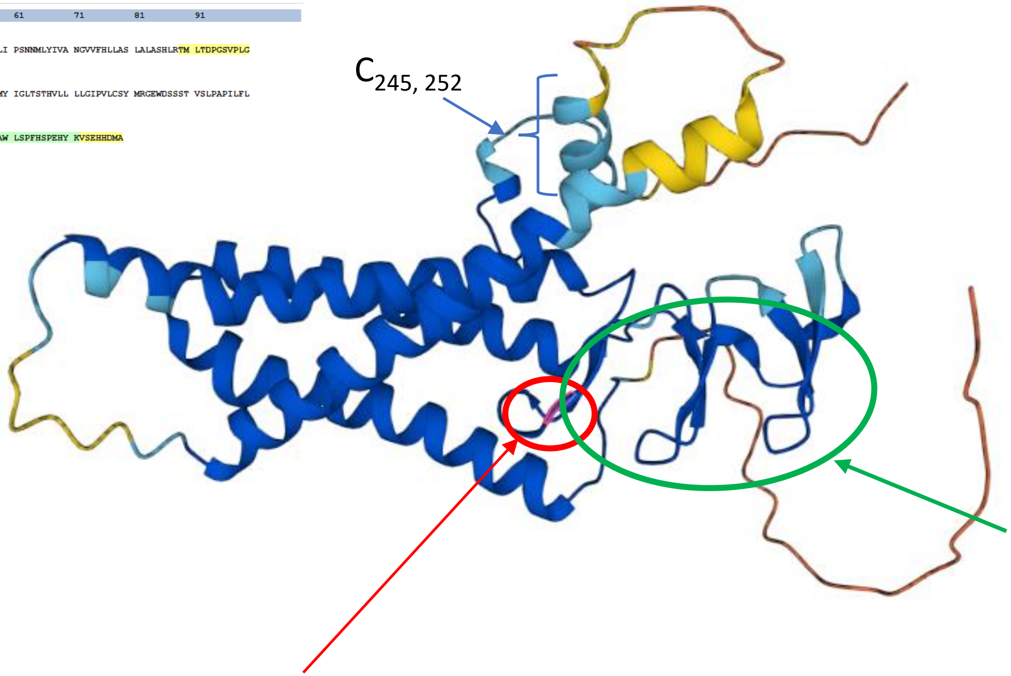

$C_{245,\ 252}$

Cysteine stack - down-stream to active site

C_{139} – active site

YCTDCHSAIPRTACHCTVCQRCIRKNDHHC$_{139}$PWINNCIG
